# Inhibition of miR-21 in glioma cells using catalytic nucleic acids

**DOI:** 10.1038/srep24516

**Published:** 2016-04-15

**Authors:** Agnieszka Belter, Katarzyna Rolle, Monika Piwecka, Agnieszka Fedoruk-Wyszomirska, Mirosława Z. Naskręt-Barciszewska, Jan Barciszewski

**Affiliations:** 1 Institute of Bioorganic Chemistry, Polish Academy of Sciences, Noskowskiego 12/14, 61-704 Poznan, Poland

## Abstract

Despite tremendous efforts worldwide, glioblastoma multiforme (GBM) remains a deadly disease for which no cure is available and prognosis is very bad. Recently, miR-21 has emerged as a key omnipotent player in carcinogenesis, including brain tumors. It is recognized as an indicator of glioma prognosis and a prosperous target for anti-tumor therapy. Here we show that rationally designed hammerhead ribozymes and DNAzymes can target miR-21 and/or its precursors. They decrease miR-21 level, and thus silence this oncomiR functions. We demonstrated that anti-miRNA catalytic nucleic acids show a novel terrific arsenal for specific and effective combat against diseases with elevated cellular miR-21 content, such as brain tumors.

Glioblastoma multiforme is the most common type of malignant primary brain tumors, accounting more than 50% of all gliomas^1^. Its localization in the brain, invasive and aggressive behavior, and extremely poor prognosis make it one of the most deadliest form of cancer^2^. Despite recent advances in surgical and radiotherapeutic techniques, it is one of the most incurable form of cancer^3^,^4^. The average prognosis for GBM patients is between 7 and 12 months^5^.

Over the past decade, a great progress in genome-wide techniques have allowed for reconstruction of genetic and molecular networks involved in tumorigenesis and provide significant insights into the mechanism of gliomas^6^,^7^,^8^. Comparative analysis of the genetic landscape and the transcriptome of GBM has shown that the gene expression profile is not a direct reflection of genetic background of glioblastoma but reflects also epigenetic alterations, such as miRNA level fluctuations^9^,^10^.

miRNAs are short non-protein coding RNAs that regulate gene expression. They are implicated in cell growth, tissue differentiation, cell proliferation, embryonic development, apoptosis and cellular signaling^11^,^12^,^13^,^14^. It is proposed that up to 90% of human transcripts are potential targets of miRNAs^15^,^16^,^17^,^18^,^19^,^20^,^21^,^22^. Therefore even slight modulation of miRNA levels can have impact on cell phenotype.

A number of studies have shown that miRNAs display precise tissue-specific expression patterns and their aberrant profile may result in substantial tissue re-organization and the development of disease^23^. Altered miRNAs expression profiles have been found so far in over 160 diseases^24^, including brain tumors^25^,^26^. It has become clear that microRNAs deregulation is implicated in different steps of the tumorigenic process, from the initiation and development to progression toward the acquisition of an invasive phenotype.

High-throughput clustering analysis of miRNAs expression revealed a specific subset of miRNAs that provide a candidate molecular signature of glioblastoma^25^. miRNAs have been showed to be altered not only in glioblastoma multiforme tissue^11^,^12^,^13^, but also in body fluids of patients^28^, in brain tumors of different grades^27^, and even subdivide high- versus low-risk patients^29^. Therefore miRNAs can be used as real-time biomarkers and possess a prognostic value for GBM survival^28^,^29^. Additionally, miRNAs represent an abundant class of targets for GBM intervention^30^.

miR-21 has been the first miRNA reported as deregulated in human glioblastoma^31^. Its expression level correlates with malignancy of tumor^25^,^32^. It is also highly expressed in other tumor types such as neuroblastoma, glioma, colorectal, lung, breast, pancreas, leukaemia, lymphoma. It is the only miRNA upregulated in all types of cancer^33^. The level of miR-21 can be used for diagnosis and prognosis of GBM^32^.

miR-21 is well known as an oncomiR and is involved in all steps of tumor development, including initiation, maintenance and survival^34^. Some cancers are dependent on miR-21, termed ‘oncomiR addiction’, in order to maintain a malignant phenotype. Taking into consideration that miR-21 is the omnipresent miRNA in human tumors, it seems that its inhibition might have significant therapeutic value and provide new choices for cancer therapy^35^.

The idea of miRNA inhibition has been born a long time ago. It has been shown that miR-21 downregulation in glioma cell lines resulted in inhibition of cell proliferation, enhanced apoptosis, decreased cell invasiveness, sensitized resistant cells to standard treatment^36^,^37^,^38^, and inhibition of tumor growth in a mouse model^39^.

A method, which causes 100% eradication of a specific miRNA is just the deleting of a miRNA gene. This method is useful for determination of miRNA function in a lab, but it is technologically difficult to use in a clinical therapy^40^,^41^. Other approaches involve blocking miRNA processing and maturation at the posttranscriptional level. Small molecule inhibitors^42^,^43^,^44^ as well as 12-mer DNAs can selectively regulate miRNA production by targeting pre-miRNA and human Dicer^45^. Antisense oligonucleotides (AS-ON) targeting mature miR-21 were used in glioblastoma cells to verify and identify their potential target sequences and to study the effects of miR-21 inhibition^46^. All of these approaches, although being very effective, have several disadvantages and the mechanism of their action is poorly understood, which prevents their testing in clinical trials^47^,^48^,^49^,^50^. Because there is a constant need for new anti-cancer remedies, other approaches such as miRNA sponges^51^, catalytic nucleic acids^52^ and miRNA supplementation^53^,^54^,^55^ are still under evaluation. Catalytic nucleic acids, both hammerhead ribozymes and DNAzymes directed against miRNA are a subject of our interest.

Ribozymes are RNA motifs with autocatalytic cleavage capability. They were found in viroids and viral satellite RNAs which are self-cleaved while replicated^56^,^57^. Although natural hammerhead ribozymes perform a single intramolecular RNA bond hydrolysis reaction *in cis*, *trans*-acting ribozymes capable of multiple turnover cleavage of RNAs have been developed^58^. At the same time, to date no DNA catalysts have not been found in nature, however DNAzymes were isolated by *in vitro* selection^59^. The highly conservative ribozyme and DNAzyme catalytic cores are flanked by arms of any sequence. This property allows for a rational design of catalytic nucleic acids for targeting of virtually any RNA. Both ribozymes and DNAzymes hydrolyze the single phosphodiester bond of RNA similarly to RNase A, giving products with a 5′-hydroxyl group and a 2′,3′-cyclic phosphate^60^,^61^. Thus, ribozymes and DNAzymes provide simple and a highly specific tool for targeting RNA of a determined sequence^62^. Ribozymes and DNAzymes are commonly used to down-regulate gene expression by hydrolyzing particular mRNAs^63^,^64^,^65^. Here we show that they may also be used to down-regulate miRNA expression. Due to their catalytic capacity, ribozymes and DNAzymes are expected to be more efficient and specific in miRNA inhibition than anti-miRNA antisense oligonucleotides^66^. Recently, antagomirzymes directed to miRNA precursors have been described^52^,^67^. However, no ribozyme activity against mature miR-21 has been shown^52^. Here, we describe for the first time the application of hammerhead ribozymes and DNAzymes to inhibit a miR-21, its precursors and therefore its function in glioma cells.

## Results

### Designing of the anti-miR-21 hammerhead ribozymes and DNAzymes

Recently, using miRNA microarrays, we analysed the miRNA expression pattern in malignant glioma tissues, tumor margins and normal human brain and found 35 miRNAs whose expression is frequently deregulated in GBM patients^25^. miR-21 is significantly upregulated miRNA in glioblastoma tissues, as well as in peritumoral areas, in comparison with normal human brain^25^. Additionally, we showed that miR-21 levels differ in low-grade gliomas (grade II), anaplastic astrocytomas and oligodendrogliomas (grade III), and glioblastoma multiforme (grade IV). It is significantly lower in grade II gliomas and in most abundant grade III tumors than in the most malignant glioblastoma multiforme^25^. Many other independent studies confirmed at consistency^5^,^68^.

Recently, to understand specificity of miRNA action, we looked for structure of mature miRNAs^69^. Using specific nucleases, T1, V1 and S1 as well as NMR, UV/Vis and CD spectroscopies, we found that not only pre-miR-21 but also mature miR-21 can adopt hairpin structure, which affects their turnover and function^69^. Considering miR-21 and it precursors as therapeutic targets, we investigated how their structures affect their accessibility for oligonucleotide binding.

Out of eight designed oligonucleotides (O1–O8) complementary to different regions of pre-miR-21, only O5 binds to G35-C41 region (pre-miR-21 loop) and forms a complex with pre-miR-21 cleaved with RNase H1 (Supplementary Fig. 1a,b). In case of mature miR-21, one can see RNA cleavage with RNase H1 in presence of each designed oligonucleotides (O1, O9–O12). Only slight differences in intensity of RNA products of reactions in the presence of oligo DNAs were observed. The lowest RNase H1 activity was observed in presence of O1 and O11. That indicated that miR-21 regions complement to oligos (A7-C13 and G11-U17) are slightly less prompt to complex oligodeoxynucleotides (Supplementary Fig. 1a,c).

Although, our results showed that pre-miR-21 hairpin has limited accessibility to oligodeoxyribonucleotides, taking into account that RNAs could be cleaved with catalytic nucleic acids not only within single but also double stranded regions^70^, we designed a set of hammerhead ribozymes and DNAzymes targeting both mature miR-21 and its precursor.

**Figure 1:**
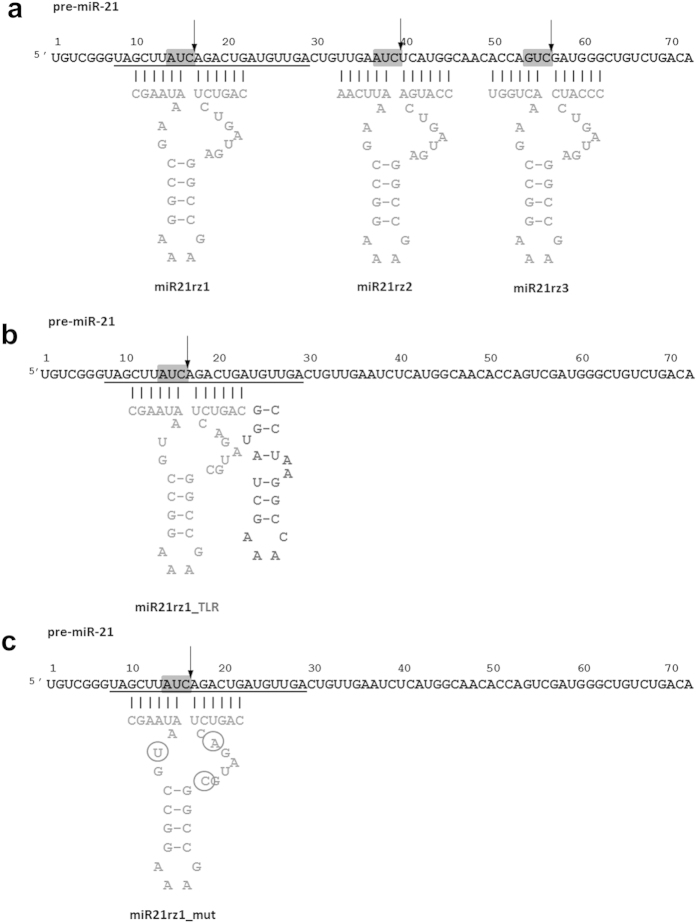
Secondary structure of anti-miR-21 ribozymes: miR21rz1, miR21rz2, miR21rz3 (**a**), miR21rz1_TLR (**b**) and mutated miR21rz1 (miR21rz1_mut) (**c**) in a complex with their substrates (pre-miR-21 or miR-21). Mature miRNA sequence is underlined, ribozyme sequence marked in grey, TLR sequence in dark grey, AUC and GUC target sites for ribozymes in grey boxes, arrows point the sites of cleavage, circles mutated nucleotides within catalytic core of miR21rz1_mut. A stands for adenosine, U – uridine, C – cytosine, G – guanosine. See also Table S1 and Fig. S3.

The hammerhead ribozymes have been designed according to the rules suggested ealier^58^,^71^,^72^. The cleavage specificity of the hammerhead ribozyme is generally described as the NUH↓ rule, where N can be any nucleotide and H cannot be G^73^. AUC and GUC sequences are the most efficiently cleaved with ribozymes thus they have been chosen as targets for anti-miR-21 ribozymes. There are two AUC and three GUC sequences within pre-miR-21, one AUC within mature miR-21. Two GUC were ignored while designing ribozymes because they are located in the vicinity of pre-miRNA ends, thus may preclude binding of ribozyme arms to target RNA. (Fig. 1, Supplementary Fig. 2, Supplementary Table 1).

**Figure 2:**
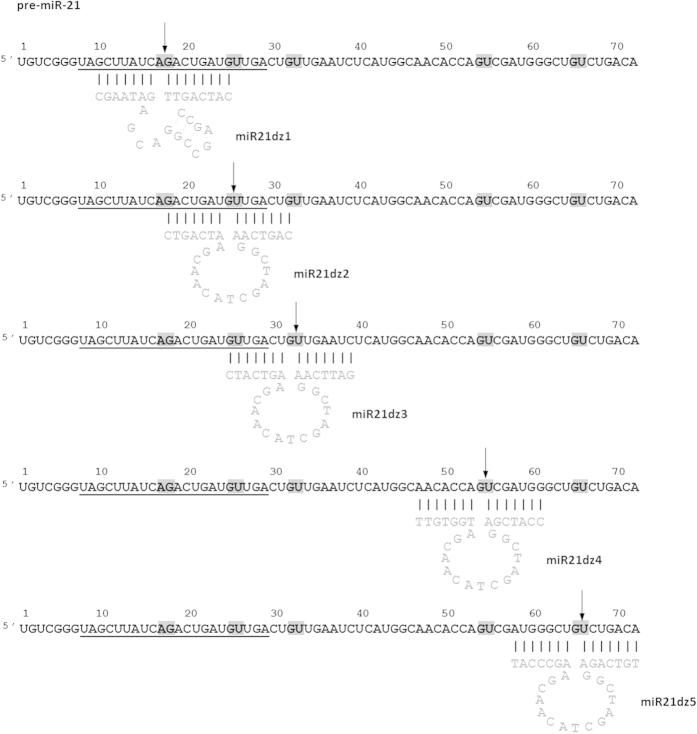
Secondary structure of anti-miR-21 DNAzymes: miR21dz1 (‘8–17’), miR21dz2, miR21dz3, miR21dz4, miR21dz5 (‘10–23’) in a complex with their substrates (pre-miR-21 or miR-21). Mature miRNA sequence is underlined, DNAzyme sequence in grey, AG and GU target sites for DNAzymes in grey squares, arrows point the sites of cleavage. A stands for adenosine, U – uridine, C – cytosine, G – guanosine. See also Table S1 and Fig. S3.

We found AUG and GUC sequences are located within hairpin stem and AUG within pre-miR-21 hairpin loop (Supplementary Fig. 2). As mentioned above, RNase H1 assay showed low availability of pre-miR-21 hairpin stem for oligonucleotides binding, but a cleavage reaction occurred in presence of oligo DNA complement to pre-miR-21 hairpin loop (G35-C41). Despite that, taking into account that nucleic acids may force the availability of other sequences^74^, ribozymes for all these sites were designed (Fig. 2). For mature miR-21, a similar efficiency of RNA cleavage by RNase H1 was observed in presence of all designed oligonucleotides complement to different regions of this miRNA. It seems that miR-21 structure is prompt for oligonucleotides invasion and suggests that it can be better therapeutic target than its precursors.

The ribozymes comprised of 34 nucleotides, including 22 nt catalytic core and 6 nt flanking complement sequence at its 3′ and 5′ hybridizing arm, were designed (Fig. 1). The catalytic domain of ribozymes was selected based on our previous experience with catalytic RNAs and well-studied hammerhead ribozyme sequence motifs^71^. The ribozymes contain two stretches of conserved nucleotides, one of the three double helices (helix II) and flanking arms (stem I and II). The ribozymes activity and specificity depends on the length of flanking arms and 6 nt arms is a balance between these two parameters^75^. The ribozymes bind to the target RNA through complementary base pairing with stems I and III. Ribozymes with their substrates form three double helices and single-stranded regions encompass the catalytic core of the ribozyme. Cleavage of the substrate RNA occurs at nonpaired residue positioned between stems I and III. The site of cleavage occurs at the 3′ site of C of sequence AUC (ribozyme miR21rz1 and miR21rz2) or GUC (ribozyme miR21rz3). Ribozyme miR21rz1 can simultaneously cleave both mature and precursor miRNA.

The limitation of catalytic RNAs is their low activity at physiological Mg^2+^ concentrations. It has been found that TSMs (tertiary stabilizing motifs) occurring in natural hammerhead ribozymes play an important role in acquiring the catalytically active conformation^76^. These so called extended of full-length ribozymes are highly active at physiological Mg^2+^ concentrations in contrast with minimal variants, which require 10 mM Mg^2+^ for efficient catalysis^77^. Previously, we showed that the TLR (tetraloop receptor) can be used as a stabilizing element that increases ribozyme catalytic activity even at low magnesium^71^. Thus TLR-extended hammerhead ribozyme, miR21rz1_TLR was designed (Fig. 1b, Supplementary Table 1). Additionally mutated ribozyme with three substitutions within catalytic core was used as a negative control for above mentioned anti-miR-21 ribozymes (Fig. 1c, Supplementary Table 1).

The DNAzymes for miR-21 were designed according to Santoro and Joyce suggestions^78^. The ‘10–23’ DNAzyme requires G(C/U), A(C/U) dinucleotides with the highest cleavage efficiency at AU and GU, whereas the ‘8–17’ deoxyribozymes solely AG as recognition sequence. AG and GU dinucleotides were chosen as cleavage sites for our ‘8–17’ and ‘10–23’ DNAzymes (Supplementary Fig. 2 and Supplementary Table 1).

The catalytic loop structure of the ‘8–17’ and ‘10–23’ deoxyribozymes contain 15 and 13 nucleotides, respectively. Like in case of hammerhead ribozymes, it is surrounded by sequence-specific flanking arms. Both types of catalytic DNA, 3′ and 5′ hybridizing arm have been designed to form 7 and 8 Watson-Crick base pairs with targets, respectively. miR21dz1-5 DNAzymes arms are complement to miR-21 precursor, miR21dz1 and miR21dz2 could recognize also mature miRNA. ‘8–17’ and ‘10–23’ deoxyribozymes have been design to cleave targets at the 5′ site of free A and G, respectively^79^.

The sequences of anti-miR-21 catalytic nucleic acids are shown in Supplementary Table 1.

### *In vitro* activity of hammerhead ribozymes and DNAzymes

**Figure 3:**
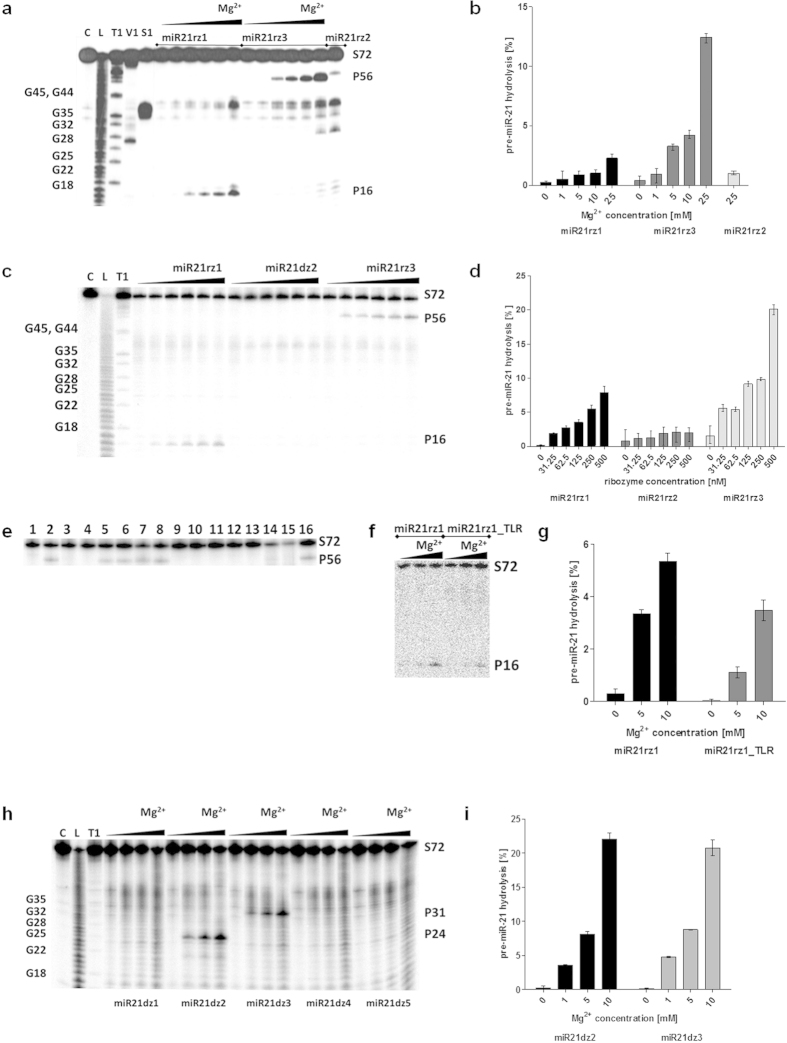
Pre-miR-21 hydrolysis by hammerhead ribozymes and DNAzymes. (**a**,**b**) Magnesium effect on RNA hydrolysis by hammerhead ribozymes. 0.1 pmole pre-miR-21 and 10 000 cpm [^32^P]pre-miR-21 was incubated with 2.5 pmol of hammerhead ribozyme and 1, 5, 10, or 25 mM MgCl_2_. For miR21rz2 only the reaction at 25 mM magnesium ions is shown. (**c**,**d**) Ribozyme:substrate ratio dependence of RNA hydrolysis by ribozymes. 0.1 pmole pre-miR-21 and 10 000 cpm of [^32^P]pre-miR-21 was incubated with 0.3125, 0.625, 1.25, 2.5 and 5 pmole ribozyme and 10 MgCl_2_. (**e**) RNA hydrolysis by ribozyme in different metal ions and molecular crowding mimicking compounds. 0.1 pmole pre-miR-21 and 10 000 cpm [^32^P]pre-miR-21 was incubated with 2.5 pmol of miR21rz3 and 10 mM MgCl_2_ (2–7, 16), 10 mM NaCl (10), 10 mM NH_4_Cl (11), 10 mM LiCl (12), KCl (13), CaCl_2_ (14), SrCl_2_ (15), 16% PEG200 (3), 16% PEG400 (4), 16% PEG 3350 (5), PEG4000 (6), 40 mM spermine (7), 40 mM spermidine (8). (**f**,**g**) Magnesium effect on RNA hydrolysis by miR21rz1 and miR21rz1_TLR. 0.1 pmole pre-miR-21 and 10 000 cpm [^32^P]pre-miR-21 was incubated with 5 pmol of miR21rz1 and miR21rz1_TLR and 10 mM MgCl_2_. (**h**,**i**) Magnesium effect on RNA hydrolysis by DNAzymes. 0.1 pmole pre-miR-21 and 10 000 cpm [^32^P]pre-miR-21 was incubated with 2.5 pmol of DNAzymes in 50 mM Tris-HCl, pH 7.5, containing 1, 5 and 10 mM MgCl_2_. As no cleavage for miR21dz1, miR21dz4 and miR21dz5 were observed they were omitted in the graph (**g**). All reactions was performed in 50 mM Tris-HCl buffer, pH 7.5, in 10 μl total reaction volume for 16 h at 37 °C. C, L, T1, V1, S1 as in the Fig. 2.

*In vitro* assays showed that miR21rz1, miR21rz2, miR21rz3 ribozymes cleave pre-miR-21 with different efficiency (Fig. 3a,d). miR21rz3 ribozyme is the most and miR21rz2 the last active one. In all cases, the efficiency of ribozymes depends on the Mg^2+^ concentration and ribozyme:substrate ratio. The *in vitro* activity of ribozymes was checked in the range from 0.5 to 50 mM Mg^2+^. The reaction took place smoothly at 50, 25, 10 and 5 mM Mg^2+^, slightly at 1 mM magnesium ions (physiological concentration), but not below.

Although originally thought that only Mg^2+^ are obligate for folding and activity of ribozymes^64^, it has been shown that a wide variety of metal ions may support their catalysis^80^. Nuclear magnetic resonance and crystallographic studies have indicated that Na^+^, Li^+^ and NH_4_^+^ facilitate tertiary structure formation, which is similar or identical to that observed in the presence of Mg^2+^ 81. Moreover, *in vitro* catalytic reaction at monovalent ions concentration of 1–4 M, in absence of magnesium ions has been observed^81^. Metal ions seems to neutralizalize the charge of RNA allowing better RNA folding, rather than playing essential chemical role in catalysis. This has led to a generally accepted model in which a variety of monovalent or divalent metal ions can function to stabilize ribozyme structure, whereas one or more divalent metal ions play a direct role in active-site chemistry^80^,^81^. Additionally, the formation of RNA active conformation, which determine its activity, is driven by water structure, which has been shown at high hydrostatic pressure and high monovalent salt concentrations^82^.

We checked weather designed ribozymes hydrolyze RNA targets in absence of Mg^2+^, at physiological concentrations of other ions and additionally in presence of molecules, which mimic cellular crowding. We showed that milimolar concentrations of monovalent (Na^+^, NH_4_^+^, Li^+^) or divalent ions (Ca^2+^, Sr^2+^) are not sufficient for ribozymes’ catalytic activity. Moreover, their activity is inhibited by polyethylene glycols of low molecule mass (200 and 400 Da), but remains almost unchanged in presence of PEG3350 and PEG4000. Spermine and spermidine at 40 mM concentration slightly increase catalytic activity of this RNA (Fig. 3e).

**Figure 4:**
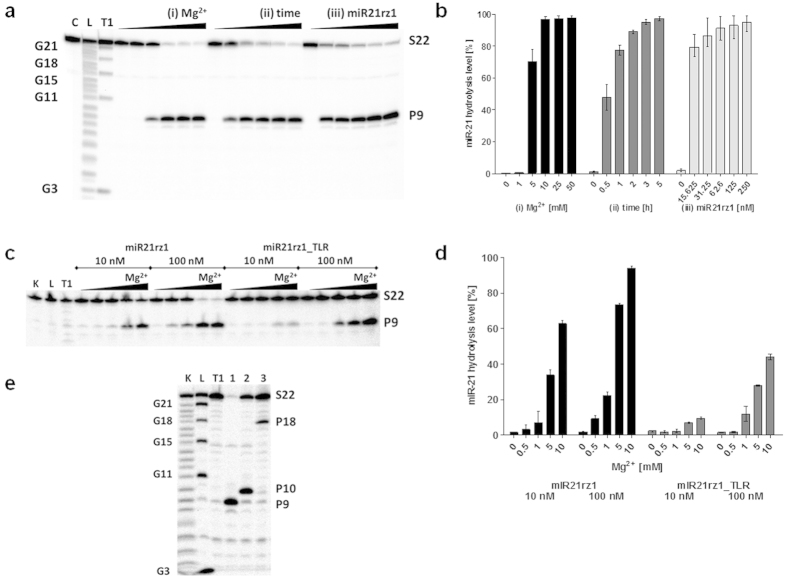
miR-21 hydrolysis by hammerhead ribozymes and DNAzymes. (**a**,**b**) Magnesium, time and ribozyme:substrate ratio dependence of RNA hydrolysis by ribozyme miR21rz1. 0.1pmole miR-21 and 10, 000 cpm [^32^P]miR-21 was incubated in 50 mM Tris-HCl, pH 7.5, buffer containing (i) 1 pmol miR21rz1 and 0, 0.5, 5, 10, 25 and 50 mM MgCl_2_ (ii) 1 pmol miR21rz1 and 10 mM MgCl_2_, (iii) 0.1, 0.3, 0.6, 1 and 2.5 pmole miR21rz1 and 10 mM MgCl_2_. Reaction were carried in in 10 μl total reaction volume at 37 °C for 1 h (i and iii) or for 0.5, 1, 2, 3, 5 h (ii). (**c**,**d**) Magnesium and ribozyme:substrate ratio dependence of RNA hydrolysis by ribozyme miR21rz1 and miR21rz1_TLR. 0.1 pmole miR-21 and 10 000 cpm of [^32^P]miR-21 was incubated with 0.1 and 1 pmole hammerhead ribozyme (miR21rz1, and miR21rz1_TLR) in 50 mM Tris-HCl, pH 7.5, buffer containing 0.5, 1, 5, 10 mM MgCl_2_ in 10 μl total reaction volume for 1 h at 37 °C. (**e**) Comparison of anty-miR-21 ribozyme and DNAzymes activity toward miR-21. 0.1 pmole miR-21 and 10 000 cpm of [^32^P]miR-21 was incubated with 1 pmole miR21rz1 (1), miR21dz1 (2) and miR21dz2 (2) in 50 mM Tris-HCl, pH 7.5, buffer containing 10 mM MgCl_2_ in 10 μl total reaction volume for 1 h at 37 °C. C, L, T1 as in the Fig. 2. Reactions were stopped with 10 μl of a loading buffer. Reaction products were separated on a 20% polyacrylamide gel with 7 M urea and quantified using the ImageQuant software.

The weakness of catalytic RNAs and DNAs is their low activity at physiological Mg^2+^ concentrations. It has been found that TLR (tetraloop receptor motif) occurring in natural hammerhead ribozymes play an important role in acquiring the catalytically active conformation^76^. These so called extended of full-length ribozymes are highly active at low Mg^2+^ in contrast with minimal variants that often require 10 mM Mg^2+^ for efficient catalysis^77^. As we identified previously, the TLR can be used as a stabilizing element that increases ribozyme catalytic activity even in low magnesium^71^, we designed and tested TLR-extended anti-miR-21 hammerhead ribozyme (miR21rz1_TLR) (Supplementary Table 1). Its activity toward pre-miR-21, same as miR21rz1, depends on Mg^2+^ concentration and ribozyme: substrate ratio. It turn out that miR21rz1_TLR hydrolyze pre-miR-21 less efficiently than the primary ribozyme (miR21rz1) (Fig. 3f,g). For example, under 50-fold excess of ribozyme over substrate, after 15 h, 3.3 and 5.3% pre-miR-21 is hydrolyzed with miR21rz1 at 5 and 10 mM Mg^2+^, respectively. For comparison, only 1.1 and 3.5% with miR21rz1_TLR. miR21rz1_TLR is also less active than miR21rz1 toward miR-21 (Fig. 4c,d). For example, at 10-fold excess of ribozyme over substrate, after 1 h, 9.19, 22.42, 73.23 and 93.96% miR-21 is hydrolyzed with miR21rz1 and 1.81, 11.99, 27.8 and 43.78% with miR21rz1_TLR at 0.5, 1, 5 and 10 mM Mg^2+^, respectively (Figs 3f,g and 4c,d).

Also, we determined DNAzymes activities against pre-miR-21 and miR-21. miR21dz2 and miR21dz3 hydrolyze pre-miR-21 in Mg^2+^ concentration-dependent manner. At 25-fold excess of DNAzymes, after 15 h, both miR21dz2 and miR21dz3 hydrolyze RNA with a similar efficiency, almost 4, 8 and 22% pre-miR-21 at 1, 5, 10 mM Mg^2+^, respectively. miR21dz1, miR21dz4 and miR21dz5 do not hydrolyze pre-miR-21 at all under tested conditions (Fig. 3h,i). Interestingly, one of them, miR21dz1 although does not hydrolyze pre-miR-21, cut efficiently mature miRNA. At 10-fold excess of DNAzyme over miR-21, at 10 mM Mg^2+^, after 1h over 60% RNA was hydrolyzed (Fig. 4e). Surprisingly, miR21dz2 although having only four nucleotides at its 5′ arm complement to miR-21 hydrolyzed mature miRNA. At 10-fold excess of miR21dz2 over substrate, and at 10 mM Mg^2+^ 15% miR-21 after 1 h was hydrolyzed (Fig. 4e). Designed DNAzymes are less active toward miR-21 than miR21rz1 ribozyme. At mentioned above conditions, miR21rz1 hydrolyze over 90% miR-21 (Fig. 4e).

The obtained results show that both ribozymes and DNAzymes are significantly more effective hydrolyze mature miR-21 than pre-miR-21, which indicates that mature miRNA seems to be better target for therapeutic intervention. Thus in further study we will focus on these catalytic nucleic acids, which hydrolyse mature miR-21. To proper and straightforward comparison of these catalytic tools we performed descent kinetic analyses. The observed cleavage rate constant (k_obs_) value were determined for ribozymes miR21rz1, miR21rz1_TLR and DNAzymes miR21dz1, miR21dz2 in the presence of 100-fold excess of ribozyme/DNAzymes over miR-21 (Table 1).

The cleavage rate constants in single turnover reaction measured at 10 mM Mg^2+^ were 0.8265 and 0.73033 min^−1^ for miR21rz1 and miR21rz1_TLR respectively. For DNAzymes in the same conditions were 4-fold lower than for ribozymes and were 0.25198 and 0.19304 min^−1^ for miR21dz1 and miR21dz2 respectively. The k_obs_ values for mentioned catalytic nucleic acids are summarized in Table 1.

Furthermore, as we noticed that both ribozymes and DNAzymes more easily hydrolyze mature miRNA than its precursor, we decided to check if the activity of catalytic nucleic acids depends on the structure of RNA target. We designed 13-nt RNA substrates complement to miR21rz1, miR21rz2 and miR21rz3, pre-miR-21_S1, pre-miR-21_S2 and pre-miR-21_S3, respectively. It is highly probable that *in vitro* they exist as single stranded molecules, thus they might be easily bound and cleaved by ribozymes. As expected all ribozymes hydrolyze short substrates with similar efficiency, which is much higher than in case of pre-miR-21 and mature miR-21 as a substrate (data not shown).

Furthermore, to verify if he structure of RNA substrate may hamper the activity of catalytic nucleic acids, we used transcript of fused pre-miR-21 and EGFP genes as substrate for ribozymes. A reporter system based on green fluorescent protein (GFP) was used (Supplementary Fig. 3). pre-miR-21 cDNA sequence was cloned into pEGFP-N3 in-frame of the EGFR protein, under the control of cytomegalovirus (CMV) promoter. HeLa and T98G cells were transfected simultaneously with pEGFP-N3 plasmid containing the pre-miR-21 sequence (pEGFP-N3-pre-miR-21) and individual ribozymes (miR21rz1, miR21rz2 and miR21rz3). Cells transfected only with pEGFP-N3-pre-miR-21 and with plasmid and non-specific ribozyme (not complement to pre-miR-21) served as controls. Ribozyme-catalyzed hydrolysis of pre-miR-21-EGFP transcript prevents synthesis of EGFP protein.

The maximum EGFP silencing is similar with all tested ribozymes and accounts 76% and 69% in HeLa and T98G cell line, respectively (Supplementary Figs 4 and 5). The lowest IC_50_ has miR21rz2, 53 and 91.2 nM in HeLa and T98G cells, respectively (Supplementary Table 2). The activity of each ribozyme is slightly higher in the T98G than the HeLa cells (Supplementary Table 2). HeLa and T98G cells have similar susceptibility of nucleic acids uptake. The transfection efficiency of both lines with fluorescein-labeled dsRNA oligomer was almost the same (~45%), with only a small predominance (2%) for T98G and HeLa cells (Supplementary Fig. 6). One can speculate that ribozymes within the cells hydrolyze both endogenous pre-miR-21, mature miR-21 and pre-miR21-EGFP transcript, thus a decrease in EGFP level and consequently fluorescence do not fully reflect a degree of hydrolysis induced by ribozymes. We postulate that the differences in silencing effect observed in each cell lines after treatment with ribozymes is a result of significantly higher miR-21 and its precursor level, in glioblastoma derived T98G cells, than in HeLa cells. In T98G cells, miR-21 and its precursors level is respectively 50 and 65% higher than in HeLa cells (Supplementary Fig. 6C).

Finally, we observed that the fused pre-miR-21-EGFP transcript is hydrolyzed with all designed ribozymes with similar efficiency. We think that the structure of pre-miR-21 being a part of fused transcript is not hairpin as in case of bare pre-miR-21, thus is more available for all designed ribozymes.

The obtained results showed that the structure of RNA substrate may hamper its cleavage with catalytic nucleic acids. Based on that we imply that mature miR-21, as easy cleaved by ribozymes and DNAzymes, is better target for therapeutic intervention than highly structured pre-miR-21.

### Activity of hammerhead ribozymes and DNAzymes in T98G cells

**Figure 5:**
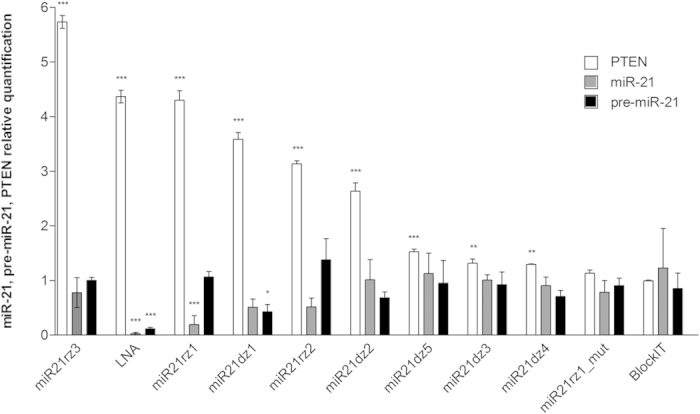
The effect of anti-miR-21 agents on the endogenous miR-21, miR-21 precursors and PTEN pools in T98G cells. T98G cells were transfected using Lipofectamine 2000 Transfection Reagent with: anti-miR-21 ribozymes (miR21rz1, miR21rz2, miR21rz3, miR21rz1_mut, 100 nM each), anti-miR-21 DNAzymes (miR21dz1, miR21dz2 and miR21dz3, miR21dz4, miR21dz5, 100 nM each) and antisense anti-miR-21 antagomir with LNA modifications (LNA, 50 nM). Non-transfected T98G cells treated only with Lipofectamine 2000 served as negative control. 24 h post-transfection cells were harvested and total RNA was isolated. RNA samples were treated with DNase I and assessed in terms of quantity and quality. They were polyadenylated and reverse-transcribed. Relative expression of miR-21 and pre-miR-21 was quantified using miR-21 forward primer and Universal Reverse Primer in qPCR using LightCycler 480 System and normalized using 18S rRNA as reference and E-Method for calculation of relative expression of miR-21 and pre-miR-21. Additionally, 24 h post-transfection cells were harvested and total protein lysate was prepared. Samples were assessed in terms of quantity and quality. Relative expression of PTEN was quantified in Western Blot and normalized using GAPDH as reference.

We check the effect of cell culture supplementation with anti-miR-21 ribozymes and DNAzymes on the level of endogenous miR-21, its precursors, both pre-miR-21 and pri-miR-21, and PTEN being a well-known, direct and functional target of miR-21^83^,^84^. The effect of different catalytic nucleic acids in not the same. Cell supplementation with miR21rz1, miR21rz2 and miR21dz1 have the biggest impact on the level of both miR-21 and PTEN (Fig. 5). MiR-21 level was decreased of 80% and 49% upon treatment with miR21rz1 and both miR21rz2 and miR21dz1, respectively. It results in PTEN upregulation of 4.3, 3.6 and 3.1 times for miR21rz1, miR21dz1 and miR21rz2. Similar effect was observed in the cells treated with LNA, which anti-miR-21 activity was previously positively verified in literature^85^,^86^. Slightly lower, 2.6 times increase of PTEN level was observed in the cells transfected with miR21dz2. As we did not observed miR-21 depletion in cells treated with this ribozyme, we think that in this case PTEN upregulation is miR-21 independent. Similarly may be in case of miR21rz3 treatment, which results in 5.7 times elevated level of PTEN, while only 20% decrease of miR-21 was observed. Other catalytic nucleic acids, miR21dz3, miR21dz4 and miR21dz5 as well as negative controls (miR21rz1_mut and Block-iT), as expected do not cause depletion of neither miR-21 or its precursor, and upregulation of PTEN expression (Fig. 5).

In conclusion, we have demonstrated that oligonucleotide enzymes, both ribozymes and DNAzymes, which recognize mature miR-21 effectively and specifically decrease miR-21 level, and thus silence their respective functions, which we showed on PTEN protein as the example of direct miR-21 target. The vast majority of designed catalytic nucleic acids which were expected do hydrolyzed pre-miRNA, but not mature miRNA are inert towards miR-21, pre-miR-21 and PTEN level in the cells. The interesting example is miR21rz2, which supplementation result in 49% depletion of miR-21 level, despite pre-miR-21 level is not lowered (even slightly increased). We postulate that although this ribozyme does not hydrolyze pre-miR-21, it recognizes pre-miR-21 hairpin loop, inhibit its maturation, which is observed as miR-21 depletion.

The obtained results provide an argument that, miR-21 is a good target for intervention with catalytic nucleic acids. Designed hammerhead ribozymes and DNAzymes decrease miRNAs level effectively and specifically, and thus silence their respective functions. This will provide means to modulate miRNA expression for therapeutic interventions.

## Discussion

Although over the past decade, significant progress in understanding mechanism of glioblastoma multiforme has been made^7^,^8^, still there is no remedy for this the most deadliest disease^2^. Thus new effective approaches in glioblastoma diagnosis and therapy are highly desired.

Recently, it have been shown that not only genetic background but also epigenetic alterations, such as miRNA level fluctuations, guide gliomas development and establishment^9^,^10^. This discovery gave new perspectives in GBM prognosis and treatment. Indeed, we found that expression of many miRNAs in human glioblastoma and glioblastoma derived cell lines is boosted and correlate with tumor grade and stage of disease^25^. Additionally, it has been shown that precise diagnosis of glioma tumors, determination of the stage of the disease, prediction of patients survival, selection of suitable treatment and monitoring its course can based on the profile of a single miRNA, such as miR-21 in postoperative tissue, as well as cerebrospinal fluid and blood serum^32^.

As, miR-21 being important in all steps of tumor development, from initiation, maintenance and survival, it is thought to be a good therapeutic target^38^. Till the idea of miRNA inhibition was born, several methods of miRNA downregulation have been investigated. miRNA blocking strategies include small molecule inhibitors to regulate miRNA precursors processing, antisense oligonucleotides to inhibit mature miRNAs, miRmasks to compete with endogenous miRNAs for mRNA binding sites, and miRNA sponge constructs to “soak up” miRNA complement to them^83^. All of these molecular tools and approaches, although being very effective, have numerous weaknesses which retard their submission to clinical trials^50^.

In this paper, we addressed the question of the possibility to regulate the level of miR-21 and its precursors with catalytic nucleic acids, hammerhead ribozymes and DNAzymes. Previously, these small nucleic acids have been successfully used to inhibit mRNA molecules to regulate their expression^58^. Since miRNA regulatory potential was observed, we decided to design catalytic RNAs and DNAs to inactivate these tiny regulatory RNAs.

We provided the hammerhead ribozymes and DNAzymes, specifically and efficiently cleaving miR-21 and/or its precursors *in vitro* and decreasing miR-21 level in the glioblastoma derived cells. The obtained results provide a strong argument that the invasion of both miR-21 and its precursors with catalytic nucleic acids is possible, thus both mature miRNA and its precursors could be a therapeutic target and anti-miR-21 ribozymes and DNAzymes are potential tools to reduce miR-21 pool in the cells. It is though that miRNAs regulate up to 90% of human transcripts and it has been shown that even slight modulation of miRNA profile has great impact on expression of numerous transcripts and thus on cell phenotype^15^,^16^,^17^,^18^,^19^,^20^,^21^,^22^. Indeed, we observed that miR-21 depletion after ribozymes and DNAzymes treatment results in significant increase of PTEN, which is a well-known, direct and functional target of miR-21^83^,^84^. Previously, it has been shown that inhibition of miR-21 and PTEN upregulation limit cellular proliferation, enhance apoptosis, decrease cell invasiveness, sensitize the chemo- or radiotherapy-resistant cells to standard treatment^34^,^35^,^36^. As we showed that anti-miR-21 catalitic nucleic acids efficiently decrease miR-21 level in the cells, we postulate that they could be possibly used in the treatment of diseases with elevated cellular miR-21 content, such as brain tumors.

Our studies gave also some general observations, which could be taken into account while designing new anti-miRNA catalytic nucleic acid tools. Since very beginning of the studies of catalytic RNAs and DNAs it was evident that the key factor for their catalytic activity is the target sequence^87^. The highest activity of these tools is associated with GUC, AUC and AG, GU, respectively for ribozymes and DNAzymes^88^. We noticed that not only sequence but also structure, which in turn depends on RNA concentration, determine efficiency of RNA hydrolysis. Mature miRNA is hydrolyzed with both ribozymes and DNAzymes significantly more efficient than its precursors. miR-21 structure is prone for oligonucleotides invasion and hydrolysis with catalytic nucleic acids, which makes it be better therapeutic target than its precursors. We proved that the structure of RNA should be taking into account while choosing therapeutic target and designing anti-miRNA tools.

Another important issue, for designing therapeutic nucleic acids is their limited delivery into the cells and susceptibility to degradation. Nucleic acid tools are much larger than conventional drugs and cannot diffuse across lipid membranes easily. Additionally, free unmodified nucleic acids are degraded in blood by nucleases and rapidly cleared from the bloodstream and would not reach the target cell^89^. Currently, three strategies to overcome these bottleneck are available: (i) chemical oligonucleotide backbone modifications, (ii) covalent conjugation with transport vehicles, and (iii) supramolecular assembly into nanosized formulation^90^. All of them improve transport of nucleic acids across cellular membranes, stabilized them against degradation, improve their binding to complementary nucleic acid target sequences and reduce the immunogenicity. The progress in this field is very fast, which allow us to believe that our and previously described catalytic nucleic acids could be used in clinic.

## Methods

### Tissue sample collection

Malignant glioma tissues and adjacent peritumoral brain tissues (glioma borders) were obtained at the time of surgery from 20 patients operated in the Department of Neurosurgery and Neurotraumatology of the Poznan University of Medical Sciences, Poland between 2010 and 2011. Tissues were flash frozen after surgery. Prior to the procedure, required donors’ approval have been obtained. Total RNA from normal human brain pooled from multiple, healthy donors and several brain regions was obtained commercially (FirstChoice^®^ Human Brain Reference RNA, Ambion).

### Cell culture

HeLa cells and T98G human glioblastoma cells were purchased from ATCC library. HeLa cells were cultured in RPMI 1640 medium supplemented with 10% fetal bovine serum, 1% antibiotic solution and 1% vitamin solution (all purchased from Sigma). T98G cells were cultured in EMEM (Eagle’s Minimum Essential Medium; ATCC) supplemented with 10% fetal bovine serum (Sigma) and 1% antibiotic solution (ATCC). Cells were grown in standard conditions (37 °C, 5% CO_2_).

### RNA isolation and quantification

Total RNA from glioblastoma multiforme, border of cancer and T98G cells was isolated using the TriPure Isolation Reagent (Roche) according to the manufacturer’s protocol. RNA samples were treated with DNase I using DNA-free DNase Treatement and Removal Reagent (Ambion) and assessed in terms of quantity and quality using Agilent 2100 Bioanalyzer and RNA 6000 Nano Kit (Agilent Technologies), agarose gel electrophoresis and NanoDrop 2000 Spectrophotometer (Thermo Scientific).

### cDNA synthesis and qRT-PCR

300 ng of each RNA sample was polyadenylated and reverse-transcribed using miRNA 1^st^-Strand cDNA Synthesis Kit (Agilent Technologies). cDNA was further diluted 1:2 with RNase-free water prior to quantification by qRT-PCR (quantitative real-time PCR). Relative expression of particular miRNAs was quantified using miRNAs forward primers (Agilent Technologies) and Universal Reverse Primer (Agilent Technologies) in quantitative RT-PCR. qRT-PCR reactions were conducted using LightCycler 480 System (Roche). miRNA expression was normalized using 18S rRNA. 25 μl reaction mixture was prepared with the DyNAmo HS SYBR Green qPCR Kit (Finnzymes) and included 1× MasterMix, 0.3× ROX reference dye, 0.3 μM of each primer, 2 μl of template cDNA and water to a final volume of 25 μl. The PCR conditions for all genes were as follows: initial denaturation (95 °C, 10 min), a four-step amplification program repeated 40–50 times [95 °C for 15 s, Tm (melting temperature) for 30 s and 72 °C for 30 s], a melting curve programme (95 °C for 1 min, 55 °C for 30 s, 55–95 °C with a heating rate of 0.1 °C/s and 95 °C for 30 s). All standard curves were generated by amplifying series of 5-fold dilutions of cDNA. The quality of PCR products was checked by an analysis of the melting curve.

### DNA and RNA synthesis

RNA substrates and ribozymes were synthesized by IBA (Germany) and Future Synthesis (Poland). DNA oligonucleotides were synthesized by Genomed S.A. (Poland). All the oligonucleotides were synthesized following standard procedures, PAGE or HPLC purified.

### RNA and DNA labelling

RNA or DNA (1 *μ*g) were labelled at the 5′-end with [*γ* -^32^P]ATP (ICN) and the T4 polynucleotide kinase (USB) at 37 °C for 45 min in 1x PNK reaction buffer. Labelled oligonucleotides were purified using denaturing polyacrylamide gel electrophoresis. The radioactive band was cut off and RNA or DNA were eluted overnight at 4 °C with an elution buffer containing 0.5 M sodium acetate and 0.1 mM EDTA, then precipitated with 2.5 vol. of 96% ethanol in the presence of 0.1 vol. of 3 M sodium acetate, pH 4.8. The pellet after 30 min of centrifugation (14000 g) was washed with 70% ethanol, centrifuged again, dried and dissolved in the RNase free water (Ambion). The quantification of the labelled RNA was performed by scintillation counting.

### RNase H1 assay

Oligonucleotides (5, 6 and 7-mers), complementary to different regions of pre-miR-21 and miR-21 were used in RNase H1 *in vitro* assay. Reactions were performed in 10 μl total volume containing 20 mM Tris-HCl, pH 7.8, 40 mM KCl, 8 mM MgCl_2_, 1 mM DTT, 1 mM pre-miR-21 or miR-21, 30 000 cpm [^32^P]-labeled pre-miR-21 or miR-21, 5 µM or 10 µM antisense DNA oligonucleotides in reactions with pre-miR-21 and 1.25, 2.5, 5 and 10 µM with miR-21 and 0.4 u *E. coli* RNase H1 (Fermentas). Samples were incubated at 37 °C for 10 min, then supplemented with 70 mM EDTA and incubated for 10 min, on ice. The reactions were stopped with 10 μl of a loading buffer (25 mM sodium citrate pH 5.0, 1 mM EDTA, 7 M urea, 0.1% bromophenol blue, 0.1% xylene cyanol). Reaction products were run along with the products of alkaline RNA hydrolysis and limited ribonuclease T1 digestion of the same RNA on a 20% denaturing (7 M urea) polyacrylamide gel and quantified using the ImageQuant software (Molecular Dynamics). To generate a sequence ladder RNA (60000 cpm), alkaline hydrolysis was performed at 95 °C for 2 min in 10 μl of reaction mixture containing 50 mM NaOH, 1 mM EDTA and 4 μg of crude tRNA from *Vigna angularis* as a carrier. In order to perform ribonuclease T1cleavages, the radiolabelled RNA (30000 cpm) was treated with 0.3 u RNase T1 (Sigma) performed in a buffer containing 20 mM sodium citrate (pH 5.0), 7 M urea and 1 mM EDTA, at 55 °C for 20 min. The cleavage yield with RNase H1 was estimated by treating the density of the control band as 100% and calculating the density of the product band as x%.

### RNA hydrolysis with ribozymes and DNAzymes *in vitro*

*Trans*-cleavage analysis of RNA with hammerhead ribozymes (miR21rz1, miR21rz2 and miR21rz3, miR21rz1_TLR, miR21rz1_mut) and DNAzymes (miR21dz1, miR21dz2, miR21dz3, miR21dz4, miR21dz5) were conducted under single-turnover conditions using an excess of the catalytic nucleic acids. All of them were analyzed under the same conditions with respect to MgCl_2_ concentration, ribozyme/target and DNAzyme/target ratio, reaction time, temperature, buffer and additional components. For single-turnover reactions, 0.1 pmol of the substrate miR-21 (22 nt) or pre-miR-21 (72 nt) was mixed with 0.1, 0.3, 0.6, 1, 2.5 or 5 pmol of ribozyme in 50 mM Tris/HCl, pH 7.5, heated at 85 °C for 2 min in a water bath, then allowed to cool slowly (overnight in case of pre-miR-21, 2 h in case of miR-21) to the reaction temperature. Cleavage reactions were initiated by addition of MgCl_2_ to a final concentration of 0.5, 1, 5, 10 or 25 mM. Total reaction volume was 10 μl. Reactions were carried out for 0.5–16 h at 37 °C and stopped with 10 μl of a loading buffer (25 mM sodium citrate pH 5.0, 1 mM EDTA, 7 M urea, 0.1% bromophenol blue, 0.1% xylene cyanol). Reaction products were separated on a 20% denaturing (7 M urea) polyacrylamide gel and quantified using the ImageQuant software (Molecular Dynamics). Cleavage yield was estimated by treating the density of the control band as 100% and calculating the density of the product band as x%.

### Hammerhead ribozyme and DNAzyme kinetic analysis

The observed rate constant (k_obs_) value were determined in the presence of 10- and 100-fold excess of ribozyme/DNAzymes which are complement to mature miR-21 (miR21rz1, miR21rz1_TLR, miR21dz1, miR21dz2). Reactions were carried out in 50 mM Tris-HCl, pH 7.5 and in presence of 10 mM MgCl_2_. Prior to reaction, probes were denatured in 85 °C for 5 min and cooled slowly (1 °C/min) down to 37 °C. Next the reactions were quenched at different time points by adding equal value of loading solution (25 mM sodium citrate, pH 5.0, 1 mM EDTA, 1M urea, 0.1% xylene cyanol and 0.1% bromophenol blue). The reaction was initiated by adding MgCl_2_ to the final concentration 10 mM. Reaction products were analyzed on denaturing 20% polyacrylamide gels. K_obs_ values were calculated by fitting to ft = 1 − exp(k_obs_t), where ft is the function cleaved at a fime t. For this purpose the Orgin Pro 8.5 software was used.

### Ribozymes and DNAzymes activity in HeLa and T98G cells

Activity of ribozymes and DNAzymes was evaluated (i) using EGFP (enhanced green fluorescent protein) reporter system, (ii) based on endogenous miR-21 and pre-miR-21 level and (iii) PTEN (phosphatidylinositol-3,4,5-trisphosphate 3-phosphatase) level (miR-21 target protein) in T98G and HeLa cells treated with ribozymes and DNAzymes. Changes in GFP fluorescence were monitored with a microscope (Leica) and plate reader, miR-21 and pre-miR-21 level was estimated by qRT-PCR, and EGFP and PTEN level by Western Blot, 24 h after transfection.

### Cells transfection

Cells were grown on 6-, 24- or 96-well multidish (Nalge Nunc International) in RPMI 1640 or EMEM medium, in standard conditions. After having reached ~80% confluence, the medium was changed for a non-supplemented counterpart medium, 1.5, 0.4 ml or 80 μl medium for 6-, 24- and 96-well dish respectively. Cell lines were transfected with the Lipofectamine^TM^ 2000 Reagent (Invitrogen) simultaneously with pEGFP-N3 plasmid containing the pre-miR-21 sequence and individual ribozymes and DNazymes directed against miR-21 or merely ribozymes or DNAzymes (without plasmid). 5, 1 and 0.25 μl Lipofectamine^TM^ 2000 Reagent were used for 6-, 24- and 96-well dish respectively. Deoxyribonucleic acids and/or ribonucleic acids and separately, Lipofectamine^TM^ were diluted in 125, 25 and 5 μl Opti-MEM (Gibco) for respectively, 6-, 24- and 96-well dish transfection and incubated for 5 min at room temperature (22 °C). Lipofectamine^TM^ mixture was added to the DNA and/or RNA mixture and incubated together for 20 min at room temperature and afterwards added to each well. The final concentration of pEGFP-N3-pre-miR-21 plasmid was 0.6 nM, ribozymes and deoxyribozymes up to 250 nM for the GFP reporter system series and up to 100 nM for others. Cells treated with antisense anti-miR-21 antagomir with LNA modifications (Exiqon, 50 nM final concentration) was used as positive control, whereas T98G/HeLa cells treated only with Lipofectamine^TM^ 2000 Reagent served as negative control. The level of EGFP protein and thus the relative activity of ribozymes were determined 24 h after transfection by (i) observation of the cell line using a Leica fluorescence microscope, (ii) fluorescence measurement using the Multi-mode BioTek Microplate Reader Synergy2 and (iii) assessment of EGFP protein levels using Western blot technique. The level of miR-21 and its precursors were determined 24 h after transfection by qRT-PCR.

### Western blot

EGFP and PTEN level of total protein of HeLa and T98G cells 24 h after transfection was estimated by Western blot with use of a ‘wet’ transfer system with the PVDF membrane and monoclonal antibodies specific against GFP, PTEN and GAPDH (glyceraldehyde 3-phosphate dehydrogenase) as reference. Cells (~2.5 × 10^6^ cells) were washed with PBS, scraped into 10 mM Tris, pH 7.5, and sonicated under the following conditions: 4 × 15 s with 1 min intervals 75% amplitude followed by centrifugation for 10 min at 4 °C and 14 000 g. Supernatant containing soluble proteins were used for Western blot analysis. Protein concentrations were measured by Nanodrop spectrophotometer. Each sample was denaturized by heating at 95 °C for 10 min. Total protein extract (50 μg of protein) were separated on 15% SDS/PAGE in a presence of protein molecular weight marker. Separated proteins were transferred (1 h, 350 mM, 100 V) on to a PVDF membrane 0.45 μm pore size (Perkin Elmer) using Western Unit (Bio-Rad Laboratories) in Towbin buffer (25 mM Tris, pH 7.5, 190 mM glycine and 20% (v/v) methanol). The membrane was blocked with 10% (w/v) dried skimmed milk powder in 1 × PBS/0.05% Tween 20 at 4 °C overnight, washed three times in 1 × PBS/0.05% Tween 20 at room temperature and incubated with the mouse monoclonal antibodies (Santa Cruz Biotechnology) against PTEN (sc-A2B1, 1:500 dilution in 3% BSA), GFP (sc-81045, 1:500 dilution in 3% BSA) and GAPDH (sc-47724, 1:1000 dilution in 3% BSA) overnight at 4 °C. After washing three times in 1 × PBS/0.05% Tween 20, the membrane was treated with the secondary biotinconjugated anti-(mouse Ig) antibody (1:5000 dilution, Sigma) for 2 h at room temperature. The membrane was washed three times and then incubated with the streptavidin–alkaline phosphatase conjugate (Amersham Biosciences) for 15 min at room temperature. After washing, membrane was developed using the BCIP (5-bromo-4-chloroindol-3-ylphosphate) NBT (Nitro Blue Tetrazolium) liquid substrate system (Sigma). Bands were quantified using ImageQuant software. The quality of proteins was checked by electrophoresis. Western blot analysis was quantified by treating the density of the control band as 100% and calculating the density of the product band as x%.

### Statistics

miRNA and it precursors level were normalized using 18S rRNA, PTEN and EGFP expression using GAPDH as reference. Error bars represent the mean ± SD of three independent experiments (biological repeats) performed in triplicate. Statistical analysis was performed using GraphPad Prism (GraphPad Software) using one-way ANOVA and Tuckey’s post hoc test. Significant differences between control and treatment groups are indicated *P < 0.05; **P < 0.01; ***P < 0.001.

## Additional Information

**How to cite this article**: Belter, A. *et al*. Inhibition of miR-21 in glioma cells using catalytic nucleic acids. *Sci. Rep*. **6**, 24516; doi: 10.1038/srep24516 (2016).

## Supplementary Material

Supplementary Information

## Figures and Tables

**Table 1:** The cleavage rate constants (*k*_*obs*_) values for anti-mature miR-21 ribozymes and DNAzymes in the presence of 100-fold excess of ribozyme/DNAzymes over miR-21 and at 10 mM Mg^2+^.

Ribozyme/DNAzyme	k_obs_	Standard error
miR21rz1	0.8265	0.01323
miR21rz1_TLR	0.73033	0.01235
miR21dz1	0.25198	0.01063
miR21dz2	0.19304	0.00992

## References

[b1] Louis D. N. Molecular pathology of malignant gliomas. Annu. Rev Pathol. 1, 97–117 (2006). 18039109 10.1146/annurev.pathol.1.110304.100043

[b2] Westermark B. Glioblastoma - a moving target. Ups J. Med. Sci. 117, 251–256 (2012). 22512247 10.3109/03009734.2012.676574 PMC3339557

[b3] Parsons D. W. . An integrated genomic analysis of human glioblastoma multiforme. Science 321, 1807–1812 (2008). 18772396 10.1126/science.1164382 PMC2820389

[b4] Fritz A. . International Classification of Diseases for Oncology, World Health Organization (2013).

[b5] Ciafre S. A. . Extensive modulation of a set of microRNAs in primary glioblastoma. Biochem. Biophys. Res. Commun. 334, 1351–1358 (2005). 16039986 10.1016/j.bbrc.2005.07.030

[b6] Yiwen J. & Lene U. On the orgin of glioma. Ups J. Med. Sci. 117, 113–121 (2012). 22348397 10.3109/03009734.2012.658976 PMC3339543

[b7] Dong H. . Integrated analysis of mutations, miRNA and mRNA expression in glioblastoma. BMC Syst. Biol. 4, 163–183 (2010). 21114830 10.1186/1752-0509-4-163 PMC3002314

[b8] Bleeker F. E., Molenaar R. J. & Leenstra S. Recent advances in the molecular understanding of glioblastoma. J. Neurooncol. 108, 11–27 (2012). 22270850 10.1007/s11060-011-0793-0 PMC3337398

[b9] Kreth S., Thon N. & Kreth F. W. Epigenetics in human gliomas. Cancer Lett. 342, 185–192 (2014). 22531315 10.1016/j.canlet.2012.04.008

[b10] Berdasco M. & Esteller M. Abberant epigenetic landscape in caner: how cellular identity goes awry. Dev. Cell. 19, 698–711 (2010). 21074720 10.1016/j.devcel.2010.10.005

[b11] Gaur A. . Characterization of microRNA expression levels and their biological correlates in human cancer cell lines. Cancer Res. 67, 2456–2468 (2007). 17363563 10.1158/0008-5472.CAN-06-2698

[b12] Godlewski J. . Targeting of the Bmi-1 oncogene/stem cell renewal factor by microRNA-128 inhibits glioma proliferation and self-renewal. Cancer Res. 68, 9125–9130 (2012). 19010882 10.1158/0008-5472.CAN-08-2629

[b13] Kim H. . Integrative genome analysis reveals an oncomir/oncogene cluster regulating glioblastoma survivorship. Proc. Natl. Acad. Sci. USA 107, 2183–2188 (2010). 20080666 10.1073/pnas.0909896107 PMC2836668

[b14] Cui Q., Yu Z., Purisima E. O. & Wang E. Principles of microRNA regulation of a human cellular signaling network. Mol. Syst. Biol. 2, 46 (2006). 16969338 10.1038/msb4100089 PMC1681519

[b15] Friedmann R. C., Farh K. K., Burge C. B. & Bartel D. P. Most mammalian mRNAs are conserved targets of microRNAs. Genome Res. 19, 92–105 (2009). 18955434 10.1101/gr.082701.108 PMC2612969

[b16] Guo H., Ingolia N. T., Weissmann J. S. & Bartel D. P. Mammalian microRNAs predominantly act to decrease target mRNA levels. Nature 466, 835–840 (2010). 20703300 10.1038/nature09267 PMC2990499

[b17] Perron M. P. & Provost P. Protein interactions and complexes in human microRNA biogenesis and function. Front Biosci. 13, 2537–2547 (2010). 17981733 10.2741/2865 PMC2901379

[b18] Bartel D. P. MicroRNAs: target recognition and regulatory function. Cell 136, 215–233 (2009). 19167326 10.1016/j.cell.2009.01.002 PMC3794896

[b19] Esquela-Kerscher A. & Slack F. J. Oncomirs - microRNAs with a role in cancer. Nat. Rev. Cancer 6, 259–269 (2006). 16557279 10.1038/nrc1840

[b20] Bartel D. P. MicroRNAs; genomics, biogenesis, mechanism, and function. Cell 116, 281–297 (2004). 14744438 10.1016/s0092-8674(04)00045-5

[b21] Kozomara A. & Griffiths-Jones S. MiRBase: integrating microRNA annotation and deep-sequencing data. Nucl. Acids Res. 39, D152–D157 (2011). 21037258 10.1093/nar/gkq1027 PMC3013655

[b22] Selbach M. . Widespread changes in protein synthesis induced by microRNAs. Nature 455, 58–63 (2008). 18668040 10.1038/nature07228

[b23] Mercer T. R., Dinger M. E., Sunkin S. M., Mehler M. F. & Mattick J. S. Specific expression of long noncoding RNAs in the mouse brain. Proc. Natl. Acad. Sci. USA 105, 716–721 (2008). 18184812 10.1073/pnas.0706729105 PMC2206602

[b24] Jiang Q. . miR2Disease: a manually curated database for microRNA deregulation in human disease. Nucl. Acids Res. 37, D98–D104 (2009). 18927107 10.1093/nar/gkn714 PMC2686559

[b25] Piwecka M. . Comprehensive analysis of microRNA expression profile in malignant glioma tissues. Mol. Oncol. S1574–7891, 00056–3 (2015). 10.1016/j.molonc.2015.03.007 PMC5528820 25864039

[b26] Wang M. . BTECH: a platform to integate genomic, transcriptomic and epigenetic alterations in brain tumors. Neuroinform 1, 59–67 (2011). 10.1007/s12021-010-9091-9 PMC3063551 21210251

[b27] Silber J. . miR-124 and miR-137 inhibit proliferation of glioblastoma multiforme cells and induce differentiation of brain tumor stem cells. BMC Med. 6, 14–31 (2008). 18577219 10.1186/1741-7015-6-14 PMC2443372

[b28] Roth P. . A specific miRNA signature in the peripheral blood of glioblastoma patients. J Neurochem. 118, 449–457 (2011). 21561454 10.1111/j.1471-4159.2011.07307.x

[b29] Srivasan S., Patric I. R. & Somasundarman K. A ten-microRNA expression signature predicts survival in glioblastoma. PLOS One 6, e17438 (2011). 21483847 10.1371/journal.pone.0017438 PMC3069027

[b30] Lacomy R. . MiR-195, miR-196b, miR-181c, miR-21 expression levels and O-6-methylguanine -DNA methyltransferase methylation status are asociated with clinical outcome in glioblastoma patients. Cancer Sci. 102, 2186–2190 (2011). 21895872 10.1111/j.1349-7006.2011.02092.x PMC11158343

[b31] Chan J. A., Krischevsky A. M. & Kosik K. S. MicroRNA-21 is an antiapoptotic factor in human glioblastoma cells. Cancer Res. 65, 6029–6033 (2005). 16024602 10.1158/0008-5472.CAN-05-0137

[b32] Hermansen S. K., Dahlrot R. H., Nielse B. S., Hansen S. & Kristensen B. W. miR-21 expression in the tumor cell compartment holds unfavorable prognostic value in gliomas. J. Neurooncol. 111, 71–81 (2013). 23104517 10.1007/s11060-012-0992-3

[b33] Volinia S. . A microRNA expression signature of human solid tumors defines cancer gene targets. Proc. Natl. Acad Sci. USA 103, 2257–2261 (2006). 16461460 10.1073/pnas.0510565103 PMC1413718

[b34] Gaur A. B., Holbeck S. L., Colburn N. H. & Israel M. A. Downregulation of Pdcd4 by mir-21 facilitates glioblastoma proliferation *in vivo*. Neuro. Oncol. 13, 580–590 (2011). 21636706 10.1093/neuonc/nor033 PMC3107097

[b35] Medina P. P., Nolde M. & Slack F. J. OncomiR addition in an *in vivo* model microRNA-21-induced pre-B-cell lymphoma. Nature 467, 86–91 (2010). 20693987 10.1038/nature09284

[b36] Zhou X. . Downregulation of miR-21 inhibits EGFR pathway and suppresses the growth of human glioblastoma cells independent of PTEN status. Lab. Invest. 90, 144–155 (2010). 20048743 10.1038/labinvest.2009.126

[b37] Corsten M. F. . MicroRNA-21 knockdown disrupts glioma growth *in vivo* and displays synergistic cytotoxicity with neural precursor cell delivered S-TRAIL in human gliomas. Cancer Res. 67, 8994–9000 (2007). 17908999 10.1158/0008-5472.CAN-07-1045

[b38] Li Y. . MicroRNA-21 targets LRRFIP1 and contributes to VM-26 resistance in glioblastoma multiforme. Brain Res. 1286, 13–18 (2009). 19559015 10.1016/j.brainres.2009.06.053

[b39] Schetter A. . MicroRNA expression profiles associated with prognosis and therapeutic outcome in colon adenocarcinoma. JAMA 299, 425–436 (2008). 18230780 10.1001/jama.299.4.425 PMC2614237

[b40] Gaveriaux-Ruff C. & Kieffer B. L. Conditional gene targeting in the mouse nervous system: insights into brain function and diseases. Pharmacol. Ther. 113, 619–634 (2007). 17289150 10.1016/j.pharmthera.2006.12.003

[b41] Park C. Y., Choi Y. S. & McManus M. T. Analysis of microRNA knockouts in mice. Hum. Mol. Genet. 19, R169–R175 (2010). 20805106 10.1093/hmg/ddq367 PMC2981466

[b42] Murata A., Fukuzumi T., Umemoto S. & Nakatani K. Xanthone derivatives as potential inhibitors of miRNA processing by human Dicer: targeting secondary structures of pre-miRNA by small molecules. Bioorg. Med. Chem. Lett. 23, 252–255 (2013). 23164709 10.1016/j.bmcl.2012.10.108

[b43] Deiters A. Small molecule modifiers of the microRNA and RNA interference pathway. The AAPS J. 12, 51–60 (2010). 19937410 10.1208/s12248-009-9159-3 PMC2811638

[b44] Gumireddy K. . Small molecule inhibitors of microRNA miR-21 function. Angew. Chem. Int. Ed. Engl. 47, 7482–7484 (2008). 18712719 10.1002/anie.200801555 PMC3428715

[b45] Kurzynska-Kokorniak A., Koralewska N., Tyczewska A., Twardowski T. & Figlerowicz M. A new short oligonucleotide-based strategy for the precursor-specific regulation on microRNA processing by Dicer. Plos One 8, e77703 (2013). 24204924 10.1371/journal.pone.0077703 PMC3812226

[b46] Kurreck J. Antisense technologies. Impreovement through novel chemical modification. Eur. J. Biochem. 270, 1628–1644 (2003). 12694176 10.1046/j.1432-1033.2003.03555.x

[b47] Dias N. & Stein C. A. Antisense oligonucleotides: basic concepts and mechanisms. Mol. Cancer Ther. 1, 347–355 (2002). 12489851

[b48] Judge A. D. . Sequence-dependent stimulation of the mammalian innate immune response by synthetic siRNA. Nat. Biotechnol. 23, 457–462 (2005). 15778705 10.1038/nbt1081

[b49] Wittrup A. & Lieberman J. Knocking down disease: a progress report on siRNA therapeutics. Nature Rev. Genet. 16, 543–552 (2015). 26281785 10.1038/nrg3978 PMC4756474

[b50] Campbell J. M., Bacon T. A. & Wickstorm E. Oligodeoxynucleozide phosphorothioate stability in subcellular extracts, culture media, sera and cerebrospinal fluid. J. Biochem. Biophys. Methods 20, 259–267 (1990). 2188993 10.1016/0165-022x(90)90084-p

[b51] Gentner B. . Stable knockdown of microRNA *in vivo* by lentiviral vectors. Nat. Methods 6, 63–66 (2009). 19043411 10.1038/nmeth.1277

[b52] Suryawanshi H., Scaria V. & Maiti S. Modulation of microRNA function by synthetic ribozymes. Mol. BioSyst. 6, 1807–1809 (2010). 20697623 10.1039/c0mb00010h

[b53] Grimm D. . Fatality in mice due to oversaturation of cellular microRNA/short RNA pathways. Nature 441, 537–541 (2006). 16724069 10.1038/nature04791

[b54] Takeshita F. . Systemic delivery of synthetic microRNA-16 inhibits the growth metastatic prostate tumors via downregulation of multiple cell-cycle genes. Mol. Ther. 18, 181–187 (2010). 19738602 10.1038/mt.2009.207 PMC2839211

[b55] Kota J. . Therapeutic microRNA delivery suppresses tumorigenesis in a murine liver cancer model. Cell 137, 1005–1017 (2009). 19524505 10.1016/j.cell.2009.04.021 PMC2722880

[b56] Symons R. H. Small catalytic RNAs. Annu. Rev. Biochem. 61, 641–671 (1992). 1497321 10.1146/annurev.bi.61.070192.003233

[b57] Uhlenbeck O. C. A small catalytic oligonucleotide. Nature 328, 596–600 (1987). 2441261 10.1038/328596a0

[b58] Haseloff J. & Gerlach W. L. Simple RNA enzymes with new and highly specific endoribonuclease activities. Nature 334, 585–591 (1988). 2457170 10.1038/334585a0

[b59] Breaker R. R. & Joyce G. F. A DNA enzyme that cleaves RNA. Chem. Biol. 1, 223–229 (1994). 9383394 10.1016/1074-5521(94)90014-0

[b60] Vaish N. K., Kore A. R. & Eckstein F. Recent developments in the hammerhead ribozyme field. Nucl. Acids Res. 26, 5237–5242 (1998). 9826743 10.1093/nar/26.23.5237 PMC148018

[b61] Wilson T. & Lilley D. M. J. RNA catalysis - is that it? RNA 21, 534–537 (2015). 25780127 10.1261/rna.049874.115 PMC4371269

[b62] Mulhbacher J., St-Pierre P. & Lafontaine D. A. Therapeutic applications of ribozymes and riboswitches. Curr. Opin. Phamacol. 10, 551–556 (2010). 10.1016/j.coph.2010.07.002 20685165

[b63] Bramlage B., Luzi E. & Eckstein F. Designing ribozymes for the inhibition of gene expression. Trends Biotechnol. 16, 434–438 (1998). 9807841 10.1016/s0167-7799(98)01236-0

[b64] Wedekind J. E. & McKay D. B. Crystallographic structure of the hammerhead ribozyme: relationship to ribozyme folding and catalysis. Annu. Rev. Biomol. Struct. 27, 475–502 (1998). 10.1146/annurev.biophys.27.1.475 9646875

[b65] Symons R. H. Plant pathogenic RNAs and RNA catalysis. Nucl. Acids Res. 25, 2683–2689 (1997). 9207012 10.1093/nar/25.14.2683 PMC146833

[b66] Woolf T. M. To cleave or not to cleave: ribozymes and antisense. Antisense Rev. Dev. 5, 227–232 (1995). 10.1089/ard.1995.5.227 8785479

[b67] Jadhav V. M., Scaria V. & Maiti S. Antagomirzymes: oligonucleotide enzymes that specifically silence microRNA function. Angew. Chemie 48, 2557–2560 (2009). 10.1002/anie.200805521 19229913

[b68] Lages E. . MicroRNA and target protein patterns reveal physiopathological features of glioma subtypes. Plos One 6, e20600 (2011). 21655185 10.1371/journal.pone.0020600 PMC3105101

[b69] Belter A. . Mature miRNAs form secondary structure, which suggests their function beyond RISC. PLos One 9, e113848 (2014). 25423301 10.1371/journal.pone.0113848 PMC4244182

[b70] Wyszko E., Barciszewska M. Z., Bald R., Erdmann V. A. & Barciszewski J. The specific hydrolysis of HIV-1 TAR RNA element with the anti-TAR hammerhead ribozyme: structural and functional implications. Int. J. Biol. Macromol. 28, 373–380 (2011). 11325424 10.1016/s0141-8130(01)00138-6

[b71] Fedoruk-Wyszomirska A., Szymanski M., Wyszko E., Barciszewska M. Z. & Barciszewski J. Highly active low magnesium hammerhead ribozyme. J. Biochem. 145, 451–459 (2009). 19124457 10.1093/jb/mvn182

[b72] McCall M. J., Hendry P. & Jennings P. A. Minimal sequence requirements for ribozyme activity. Proc. Natl. Acad. Sci. USA 89, 5710–5714 (1992). 1631050 10.1073/pnas.89.13.5710 PMC49366

[b73] Amarzguioui M. & Prydz H. Hammerhead ribozyme design and application. Cell Mol. Life Sci. 54, 1175–1202 (1998). 9849614 10.1007/s000180050247 PMC11147389

[b74] Serikov R. . Mechanism of antisense oligonucleotide interaction with natural RNAs. J. Biomol. Struct. Dyn. 29, 50 (2011). 10.1080/073911011010524987 21696224

[b75] Kurreck J., Bieber B., Jahnel R. & Erdmann V. A. Comparative study of DNA enzymes and ribozymes against the same full-length messenger RNA and the vanilloid receptor subtype I. J. Biol. Chem. 277, 7099–7107 (2002). 11751899 10.1074/jbc.M107206200

[b76] Khorova A., Lescoute A., Westhof E. & Jayasena S. D. Sequence elements outside the hammerhead ribozyme catalytic core enable intracellular activity. Nat. Struct. Biol. 10, 708–712 (2003). 12881719 10.1038/nsb959

[b77] Saksmeprome V., Roychowdhury-Saha M., Jayasena S., Khorova A. & Burke D. H. Artificial tertiary motifs stabilize trans-cleaving hammerhead ribozyme under conditions of submilimolar divalent ions and high temperatures. RNA 10, 1916–1924 (2004). 15547137 10.1261/rna.7159504 PMC1370680

[b78] Santoro S. W. & Joyce G. F. A general purpose RNA-cleavage DNA enzyme. Proc. Natl. Acad. Sci. USA 94, 4262–4266 (1997). 9113977 10.1073/pnas.94.9.4262 PMC20710

[b79] Santoro S. W. & Joyce G. F. Mechanism and utility of an RNA-cleavage enzyme. Biochemistry 37, 13330–13342 (1998). 9748341 10.1021/bi9812221

[b80] Johnson-Buck A. E., McDowell S. E. & Walter N. G. Metal ions: supporting actions in the playbook of small ribozymes. Met. Ions Life Sci. 9, 175–196 (2011). 22010272 10.1039/9781849732512-00175 PMC3365584

[b81] Murray J. B. . The hammerhead, hairpin and vs. ribozymes are catalytically proficient in monovalent cations alone. Chem. Biol. 5, 587–595 (1998). 9818150 10.1016/s1074-5521(98)90116-8

[b82] Giel-Pietraszuk M., Fedoruk-Wyszomirska A. & Barciszewski J. Effect of high hydrostatic pressure on hydratation and activity of ribozymes. Mol. Biol. Rep. 37, 3713–3719 (2010). 20204525 10.1007/s11033-010-0024-3

[b83] Xu L. F. . MicroRNA-21 (miR-21) regulates cellular proliferation, invasion, migration, and apoptosis by targeting PTEN, RECK and Bcl-2 in lung squamous carcinoma. PLos One 9, e103698 (2014). 25084400 10.1371/journal.pone.0103698 PMC4118890

[b84] Meng F. . MicroRNA-21 regulates expression of the PTEN tumor suppressor gene in human hepatocellular cancer. Gastroenterology 133, 647–658 (2007). 17681183 10.1053/j.gastro.2007.05.022 PMC4285346

[b85] Zhou X. . Downregulation of miR-21 inhibits EGFR pathway and suppresses the growth of human glioblastoma cells independent of PTEN status. Lab. Invest. 90, 144–155 (2010). 20048743 10.1038/labinvest.2009.126

[b86] Wang T. T. . MiR-21 modulates hTERT through a STAT3-dependent manner on glioblastoma cell growth. CNS Neurosci. Ther. 18, 722–728 (2012). 22709411 10.1111/j.1755-5949.2012.00349.x PMC6493453

[b87] Garzon R., Marcucci G. & Croce C. M. Targeting microRNAs in cancer: rationale, strategies and challenges. Nat. Rev. Drug Discov. 9, 775–789 (2010). 20885409 10.1038/nrd3179 PMC3904431

[b88] Sun L. Q., Cairns M. J., Saravolac E. G., Baker A. & Gerlach W. L. Catalytic nucleic acids: from lab to applications. Pharmacol. Rev. 52, 325–347 (2000). 10977866

[b89] Lachelt U. & Wagner E. Nucleic acid therapeutics using polyplexes: a journey of 50 years (and beyond). Chem. Rev. 115, 11043–11078 (2015). 25872804 10.1021/cr5006793

[b90] Wagner E. Biomaterials in RNAi therapeutics: Quo Vadis? Biomater. Sci. 1, 804–809 (2013). 10.1039/c3bm60071h 32481926

