# Supplementary material for: Inhibition of miR-21 in glioma cells using catalytic nucleic acids
Source: Sci Rep. 2016 Apr 15;6:24516. doi: 10.1038/srep24516 (PMC4832220; doi:10.1038/srep24516)
Supplement: Supplementary Information [file srep24516-s1.pdf]

SUPPLEMENTARY MATERIAL

Title: Inhibition of miR-21 in glioma cells using catalytic nucleic acids

Authors: Belter Agnieszka[1], Rolle Katarzyna[1], Piwecka Monika[1], Fedoruk-Wyszomirska A, Naskręt-Barciszewska Miroslawa Zofia, Barciszewski Jan[2]

Institute of Bioorganic Chemistry, Polish Academy of Sciences, Noskowskiego 12/14, 61-704 Poznan, Poland

[1]These authors are co-first authors on this work

[2]corresponding author, Jan.Barciszewski@man.poznan.pl

SUPPLEMENTARY TABLES

Supplementary Table 1.

Sequence of anti-miR-21 catalytic nucleic acids. Catalytic loop sequence of ribozymes and DNAzymes are underlined, TLR sequence double-underlined.

Type of catalytic nucleic acid	Name	Sequence	Target
'Hammerhead' ribozyme	miR21rz1	5'CAGUCUCUGAUGAGGCCGAAAGGCCGAAAUAAGC	pre-miR-21, miR-21
	miR21rz2	5'CCAUGACUGAUGAGGCCGAAAGGCCGAAAUUCAA	pre-miR-21
	miR21rz3	5'CCCAUCCUGAUGAGGCCGAAAGGCCGAAACUGGU	pre-miR-21
TLR-extended 'hammerhead' ribozyme	miR21rz1_TLR	5'CCUAAGGCCAAAGCUAUGGCAGUCUCUGAUGAGGCCGAAAGGCCGAAAUAAGC	pre-miR-21, miR-21
Mutated 'hammerhead' ribozyme	miR21rz1_mut	5'CAGUCUCAGAUGCGGCCGAAAGGCCGUAAUAAGC	pre-miR-21, miR-21
'hammerhead' ribozyme	TARrz	5'GGGCUCACUGAUGACUCCGGUAGGACGAAAUCUGCC	negative control for anty-miR-21 ribozymes and DNAzymes
'8-17' DNAzyme	miR21dz1	5'CATCAGTTCCGAGCCGGACGAGATAAGC	pre-miR-21, miR-21
'10-23' DNAzyme	miR21dz2	5'CAGTCAAGGCTAGCTACAACGAATCAGTC	pre-miR-21, miR-21
	miR21dz3	5'GATTCAAGGCTAGCTACAACGAAGTCAAC	pre-miR-21
	miR21dz4	5'CCATCGAGGCTAGCTACAACGATGGTGTT	pre-miR-21
	miR21dz5	5'TGTCAGAGGCTAGCTACAACGAAGCCCAT	pre-miR-21

Supplementary Table 2.

IC_{50} values of ribozymes miR21rz1, miR21rz2, miR21rz3 in T98G and HeLa cell lines transfected with pEGFP-N3-(pre-miR-21).

IC_{50} values were obtained by measurement of fluorescence emitted by cells 24 h after transfection with pEGFP-N3-(pre-miR-21) plasmid. Calculations and statistics were performed in the GraphPadPrism.

	miR21rz1	miR21rz2	miR21rz3
IC_{50} (T98G)	115.5 nM	91.2 nM	99.2 nM
IC_{50} (HeLa)	60.2 nM	53.0 nM	69.2 nM

SUPPLEMENTARY FIGURES

a

```
O8                                                   3’ CAGCTA
O7                                              3’ GTGGT
O6                                   3’ AGAGTA
O5                                3’ CTTAGAG
O4                             3’ CAACTT
O3                        3’ ACTGA
O2                 3’ GACTAC
O1           3’ TAGTCTG                                          pre-miR-21
5’ UGUCGGGUAGCUUAUCAGACUGAUGUUGACUGUUGAAUCUCAUGGCAACACCAGUCGAUGGGCUGUCUGACA 3’
O9        3’CGAATAG
O1           3’ TAGTCTG
O10            3’ GTCTGAC
O11              3’ CTGACTA
O12                  3’ CTACAAC
```

b

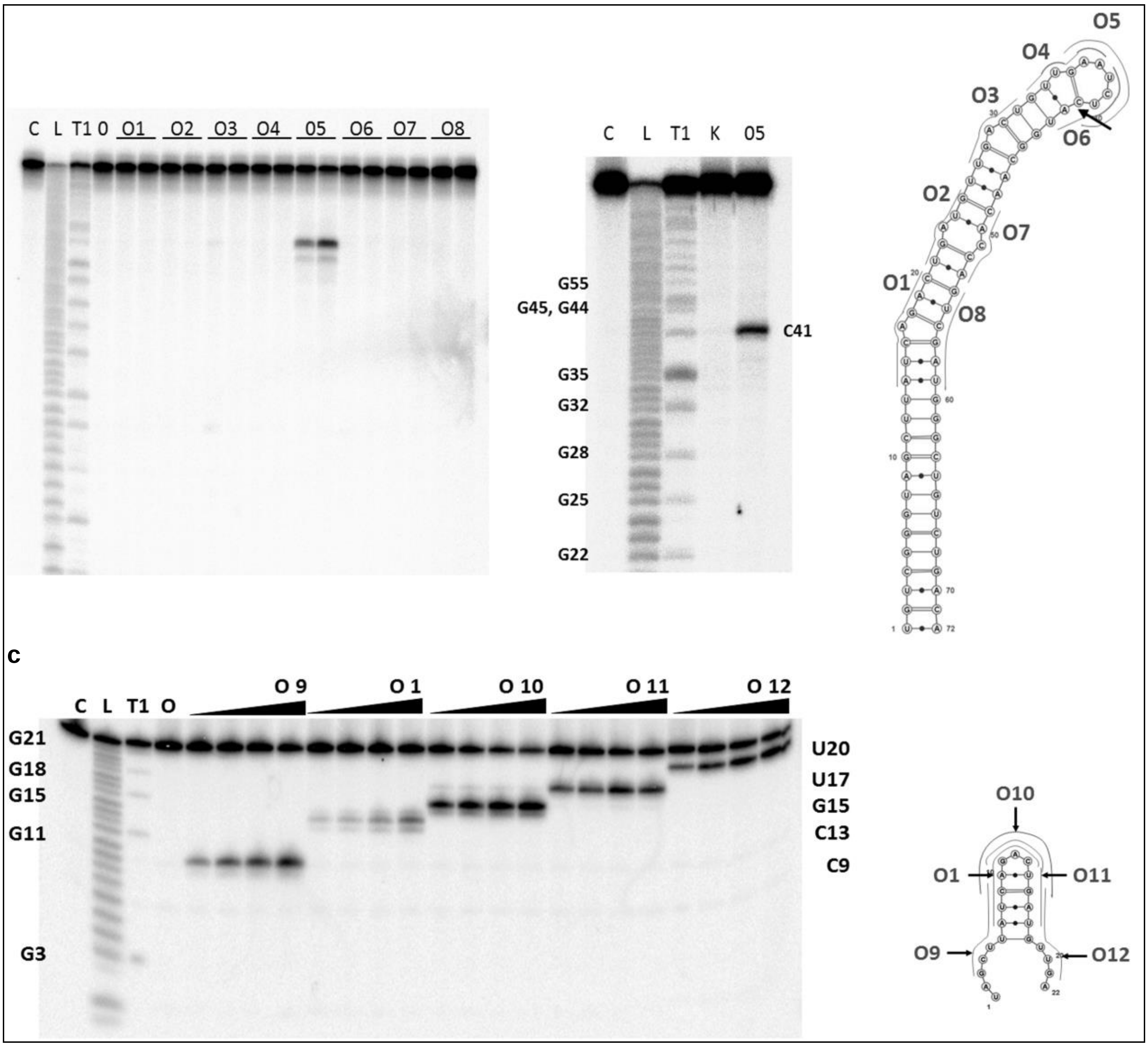

Supplementary Figure 1.

Mapping of the accessible sites of pre-miR-21 and miR-21 by RNase H1 assay.

The sequences of oligodeoxyribonucleotides (O1-O12) complement to different regions of miR-21 and pre-miR-21 used in RNase H1 assay (**a**). Sequence of oligo DNA are marked in grey, of miR-21 underlined.

Cleavage patterns obtained for limited hydrolysis of 5'-end labeled pre-miR-21 with 5 μM or 10 μM antisense oligonucleotides and RNase H1 (0.04 u/μl) (**b**) and miR-21 with 0, 1.25, 2.5, 5 or 10 μM oligonucleotides (O1, O9-O12) and RNase H1 (0.04 u/μl) (**c**). Lanes: C - reaction control; L – OH ladder; T1 - limited hydrolysis with RNase T1 (0.025 u/μl) in denaturing condition, 0 - control sample, without oligodeoxynucleotide, O1-O12 – reactions with different antisense oligonucleotides and RNase H1.

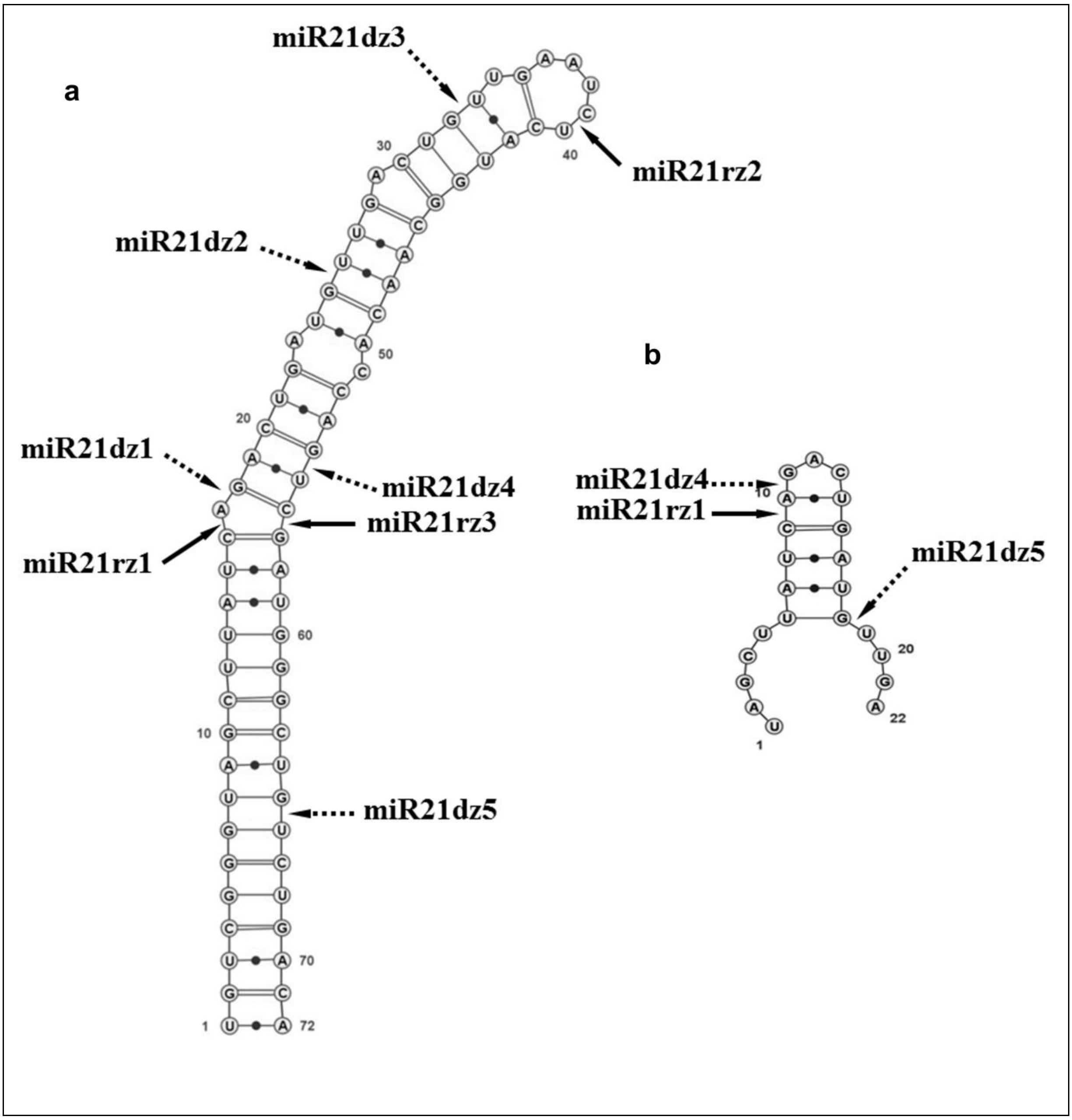

Supplementary Figure 2.

Secondary structure of pre-miR-21 (**a**) and miR-21 (**b**) and the sites of their cleavage with hammerhead ribozymes (miR21rz1-3) and DNAzymes (miR21dz1-5) pointed with solid and dashed lines, respectively.

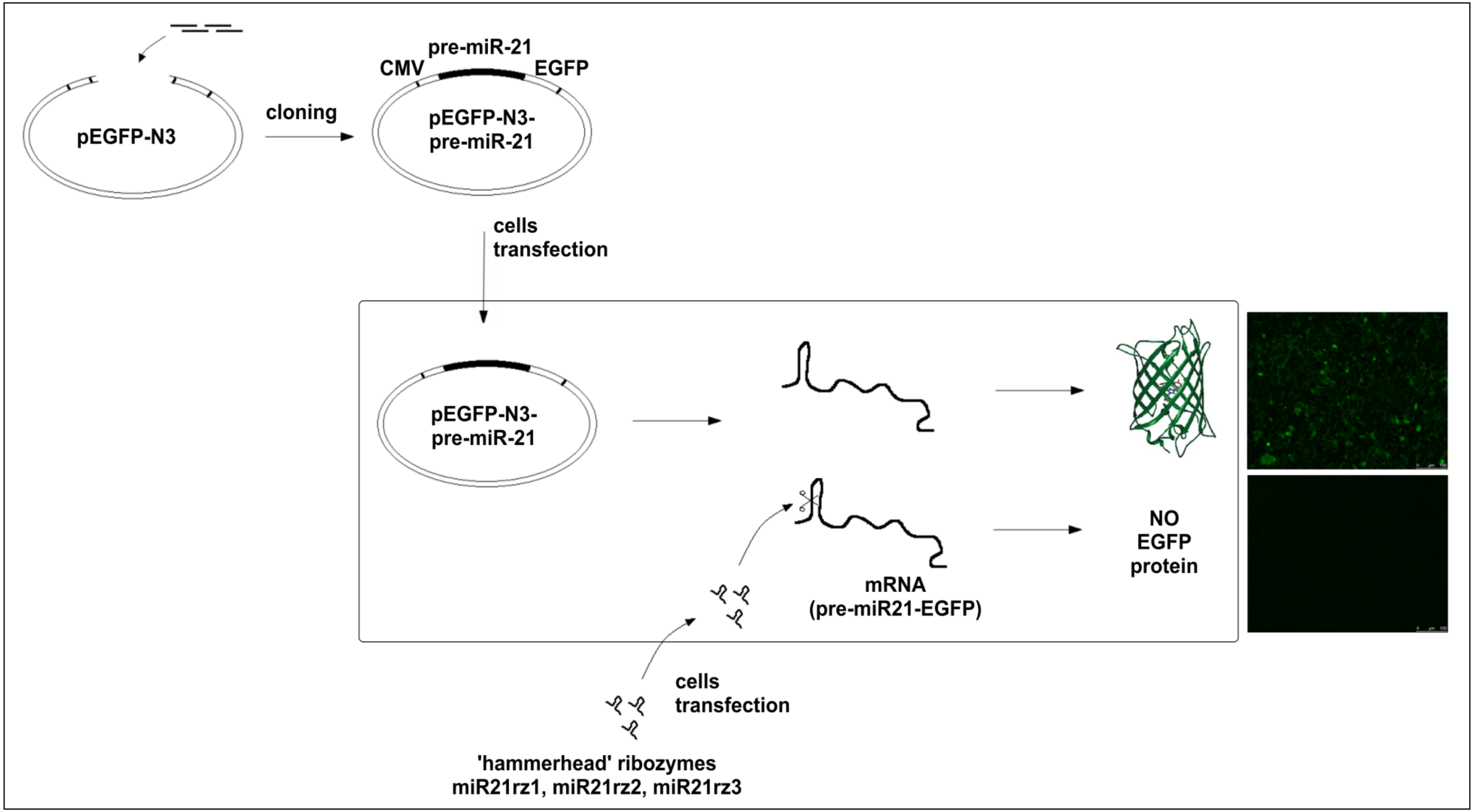

Supplementary Figure 3.

Scheme of EGFP-based assay to evaluate hammerhead ribozyme activity.

pre-miR-21 cDNA sequence was cloned into pEGFP-N3 in-frame with the EGFR protein, under the control of cytomegalovirus (CMV) promoter. Cell lines were transfected simultaneously with pEGFP-N3 plasmid containing the pre-miR-21 sequence (pEGFP-N3-pre-miR-21) and individual ribozymes directed against pre-miR-21: miR21rz1, miR21rz2 and miR21rz3. Ribozyme-catalyzed hydrolysis of the transcript comprising the pre-miR-21 and EGFP sequence prevents EGFP synthesis observed as a decrease in fluorescence in ribozyme-treated cells relative to control cells. The ribozymes activity was evaluated by measuring the level of fluorescence and by analysis of the EGFP protein level.

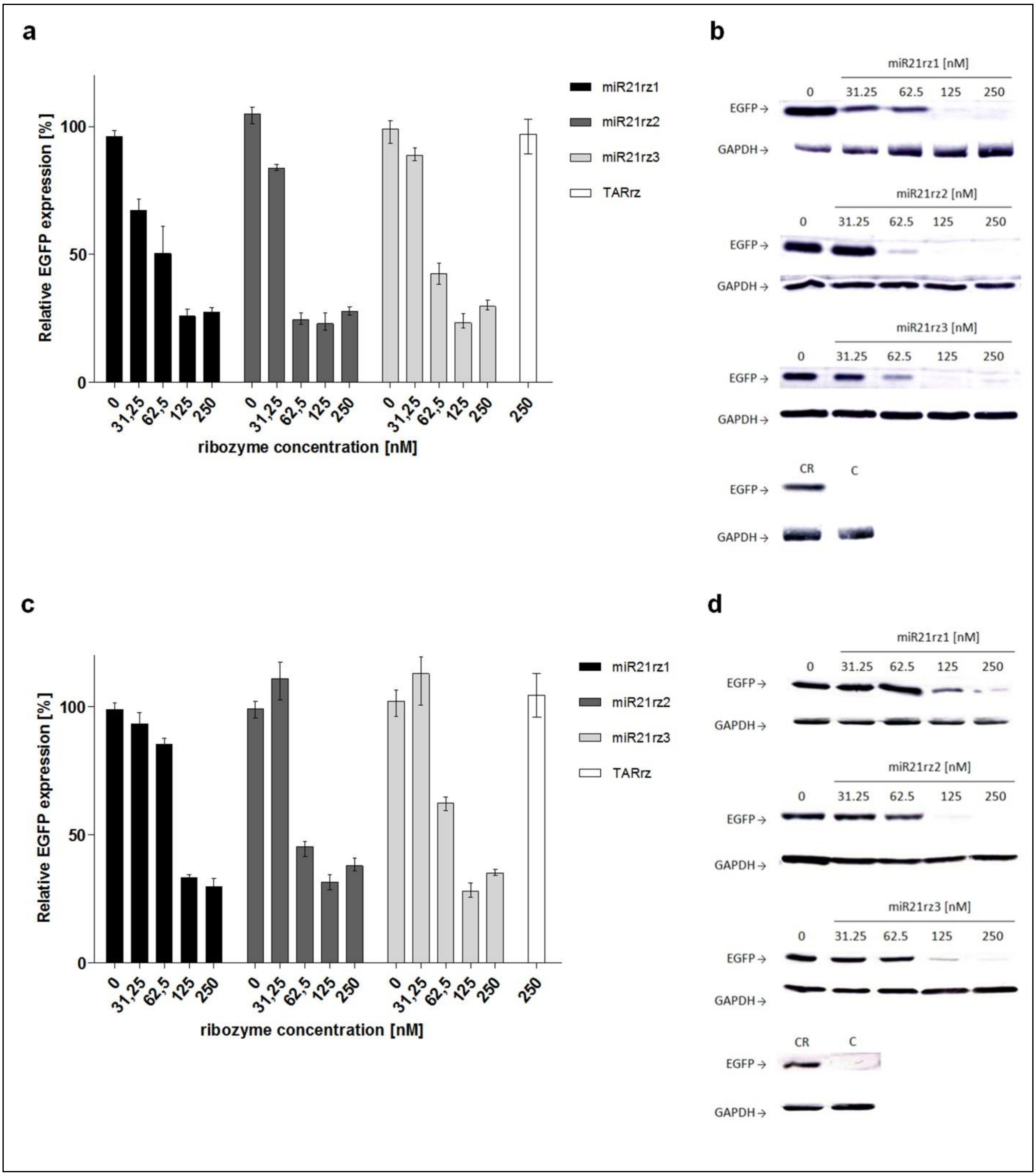

Supplementary Figure 4.

GFP expression in HeLa (**A, B**) and T98G (**C, D**) cells transfected with pEGFP-N3-(pre-miR-21) after 24 h incubation with different concentrations of hammerhead ribozymes.

a, c. The fluorescence of EGFP protein in dependence of different ribozyme concentrations.

b, d. Western blot analysis showing the level of EGFP protein level in dependence of different ribozyme concentrations.

C – reaction control (cells treated only with Lipofectamine 2000), CR – cells transfected with control ribozyme (TARrz). See also Supplementary Figures 3 and 5.

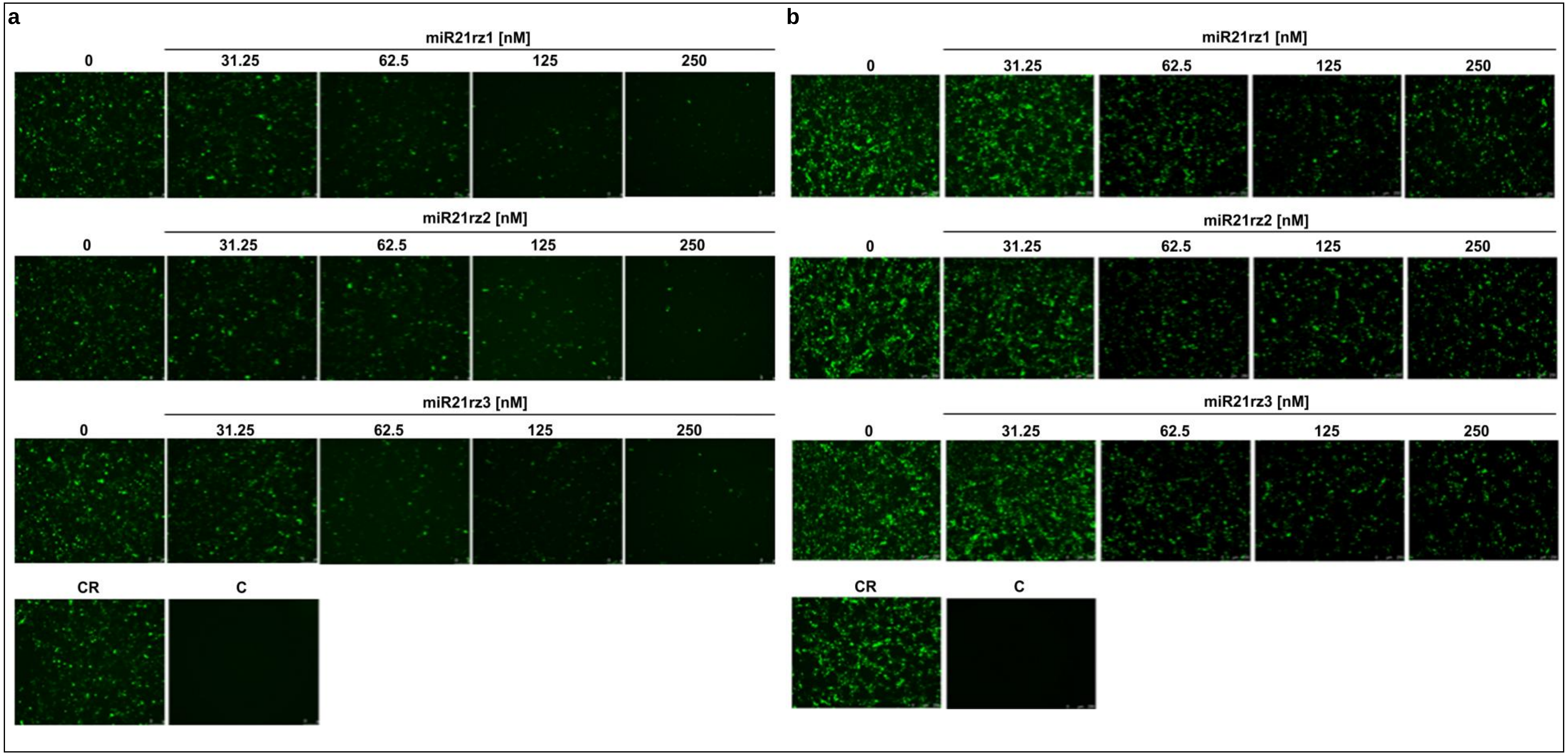

Supplementary Figure 5.

Leica fluorescent microscope images of HeLa (**a**) and T98G (**b**) cells 24 h after co-transfection with pEGFP-N3-(pre-miR-21) and ribozymes miR21rz1, miR21rz2, miR21rz3 at different concentrations (31.25, 62.5, 125, 250 nM).

C – reaction control (cells treated only with Lipofectamine 2000), CR – cells transfected with control ribozyme (TARrz).

See also Supplementary Figures 3 and 4.

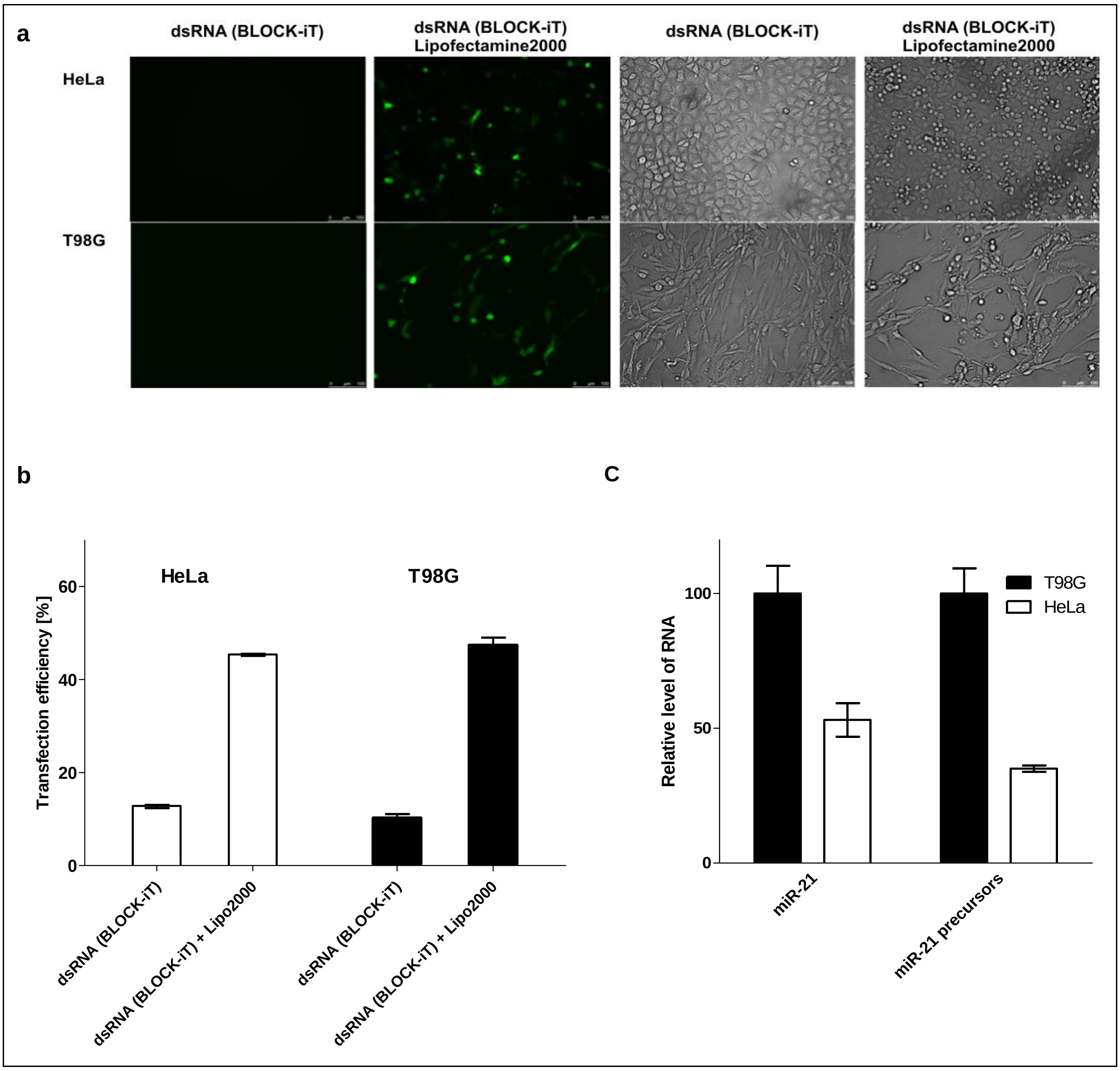

Supplementary Figure 6.

Comparison of transfection efficiency of HeLa and T98G cells with fluorescein-labeled dsRNA oligomer (BLOCK-iT) (**a, b**) and relative level of miR-21 and its precursors in HeLa and T98G cells (**c**).

~70 % confluent HeLa and T98G cells were transfected with BLOCK-iT at 30 nM final concentration. The transfection efficiency was assess 24 h after transfection based on the cells observation using a Leica fluorescence microscope (**a**) and fluorescence measurement using Multi-mode BioTek Microplate Reader Synergy2 (**b**). The efficiency of transfection was estimated with or without Lipofectamine 2000 presence. Relative level of miR-21 and its precursors were determined using qPCR (**c**). MiR-21 and its precursors level in T98G cells were treat as 100 %.
